# MYH1 deficiency disrupts outer hair cell electromotility, resulting in hearing loss

**DOI:** 10.1038/s12276-024-01338-4

**Published:** 2024-11-01

**Authors:** Jinsei Jung, Sun Young Joo, Hyehyun Min, Jae Won Roh, Kyung Ah Kim, Ji-Hyun Ma, John Hoon Rim, Jung Ah Kim, Se Jin Kim, Seung Hyun Jang, Young Ik Koh, Hye-Youn Kim, Ho Lee, Byoung Choul Kim, Heon Yung Gee, Jinwoong Bok, Jae Young Choi, Je Kyung Seong

**Affiliations:** 1 Department of Otorhinolaryngology, Brain Korea 21 PLUS Project for Medical Sciences, Yonsei University College of Medicine, Seoul, Republic of Korea; 2 Institute for Lee Won Sang Yonsei Ear Science, Seoul, Republic of Korea; 3 Department of Pharmacology, Brain Korea 21 PLUS Project for Medical Sciences, Yonsei University College of Medicine, Seoul, Republic of Korea; 4 Department of Anatomy, Brain Korea 21 PLUS Project for Medical Sciences, Yonsei University College of Medicine, Seoul, Republic of Korea; 5 Woo Choo Lee Institute for Precision Drug Development, Seoul, Republic of Korea; 6 Department of Nanobioengineering, Incheon National University, Incheon, Korea; 7 Graduate School of Cancer Science and Policy, National Cancer Center, Goyang-si, Republic of Korea; 8 Korea Mouse Phenotyping Center, Seoul National University, Seoul, Republic of Korea; 9 Laboratory of Developmental Biology and Genomics, BK21 Program Plus for Advanced Veterinary Science, Research Institute for Veterinary Science, College of Veterinary Medicine, Seoul National University, Seoul, Republic of Korea

**Keywords:** Peripheral neuropathies, Functional genomics, Mutation

## Abstract

*Myh1* is a mouse deafness gene with an unknown function in the auditory system. Hearing loss in *Myh1*-knockout mice is characterized by an elevated threshold for the auditory brainstem response and the absence of a threshold for distortion product otoacoustic emission. Here, we investigated the role of MYH1 in outer hair cells (OHCs), crucial structures in the organ of Corti responsible for regulating cochlear amplification. Direct whole-cell voltage-clamp recordings of OHCs revealed that prestin activity was lower in *Myh1*-knockout mice than in wild-type mice, indicating abnormal OHC electromotility. We analyzed whole-exome sequencing data from 437 patients with hearing loss of unknown genetic causes and identified biallelic missense variants of *MYH1* in five unrelated families. Hearing loss in individuals harboring biallelic *MYH1* variants was non-progressive, with an onset ranging from congenital to childhood. Three of five individuals with *MYH1* variants displayed osteopenia. Structural prediction by AlphaFold2 followed by molecular dynamic simulations revealed that the identified variants presented structural abnormalities compared with wild-type MYH1. In a heterogeneous overexpression system, *MYH1* variants, particularly those in the head domain, abolished MYH1 functions, such as by increasing prestin activity and modulating the membrane traction force. Overall, our findings suggest an essential function of MYH1 in OHCs, as observed in Myh1-deficient mice, and provide genetic evidence linking biallelic *MYH1* variants to autosomal recessive hearing loss in humans.

## MYH1 gene variants linked to non-progressive hearing loss

Hearing loss is a major health problem affecting many people globally, with a large part due to genes. Researchers studied the MYH1 gene, which was previously associated with hearing in mice but not in humans. The team studied the hearing in mice without the MYH1 gene and looked at genetic data from people with hearing loss. They found mice without the MYH1 gene had hearing problems and impaired hair cell function. They also found several people with changes in the MYH1 gene, providing the first proof of its role in human hearing loss. Research shows how changes in MYH1 affect the function of cells in the inner ear, causing hearing problems in both mice and humans. They hope their work will lead to improved diagnostic tools and treatments for hearing loss.

This summary was initially drafted using artificial intelligence, then revised and fact-checked by the author.

## Introduction

Hearing loss (HL) is a common sensory disorder affecting an estimated 1–2 of every 1000 newborns in the populations^1,2^. Hearing loss either occurs as an isolated condition (nonsyndromic, 70%) or presents with additional phenotypes (syndromic, 30%)^3^. Almost half of all congenital hearing loss cases have a genetic basis. Nonsyndromic hearing loss (NSHL), which accounts for 70% of genetic hearing loss, is associated with 150 loci (DFNA, DFNB, and DFNX) and more than 124 genes (Hereditary Hearing Loss Homepage; refer to Web Sources)^4,5^. Although several genes are associated with hearing loss, the genetic causes of ~60% of hearing loss cases remain unidentified, particularly those occurring in childhood and young adulthood^6–8^.

Large-scale mouse phenotype screening studies have revealed that at least 450 genes are required for murine hearing function^9,10^, suggesting that a multitude of additional genes may be linked to hearing loss in humans. These studies identified 261 deafness genes in mice that have not been detected in human patients and whose roles in the auditory system have not been elucidated, making them excellent candidates for deafness genes in humans.

*Myh1*, which encodes myosin heavy chain 1, is a mouse deafness gene and constitutes a crucial component of myosin II, a hexameric actin-based motor protein that transforms chemical energy into mechanical force through ATP hydrolysis. Within eukaryotic cells, MYH1 is one of the two myosin heavy chains in myosin II and plays a pivotal role in various motility-related processes^11^. Our research focused on elucidating the involvement of MYH1 in the auditory system and providing evidence on how MYH1 deficiency leads to hearing loss.

Prestin, a transmembrane motor protein located in the outer hair cells (OHCs) of the cochlea, promotes the electromotility of OHCs in the auditory system^12–14^. Prestin is critical for the remarkable sensitivity and frequency selectivity of the auditory pathway^15^. Prestin belongs to the SLC26 family of anion transporters but uniquely functions as a motor protein, driving the rapid electromotile response of OHCs^16,17^. This electromotility is essential for the amplification of sound vibrations, thereby greatly enhancing auditory capabilities. Mutations in the gene encoding *Prestin* are linked to hearing impairments, underscoring its crucial role^18^. However, little is known about the factors that regulate or impact the activity of prestin, which may be important in hearing.

In this study, we explored the role of Myh1 in OHCs, focusing on cellular motility, using *Myh1*-KO mice and assessing its association with prestin. Additionally, we presented five unrelated human cases segregating with biallelic missense variants of *MYH1*, providing the first genetic evidence in humans.

## Materials and methods

### Audiometric characterization of *Myh1*-knockout mice

*C57BL/6N-Myh1*^*tm1b(KOMP)Wtsi*^*/JMmucd* mice were purchased from the Mutant Mouse Resource and Research Center (MMRRC; Davis, CA, USA; Cat. # 048699-UCD) and designated *Myh1*^−*/*−^ mice. Auditory brainstem response (ABR) thresholds were measured in a soundproof chamber using Tucker-Davis Technologies (TDT) RZ6 digital signal processing hardware and BioSigRZ software (Alachua, FL, USA). Subdermal needles (electrodes) were positioned at the vertex and ventrolateral to the right and left ears of anesthetized mice. A calibrated click stimulus (10 µs duration) or tone burst stimuli (5 ms duration) were produced at 4, 6, 8, 10, 12, 18, 24, 30, and 42 kHz using SigGenRZ software and an RZ6 digital signal processor and were delivered to the ear canal by a multifield 1 (MF1) magnetic speaker (TDT). The stimulus intensity was increased from 10 to 95 dB SPL in 5 dB steps. The ABR signals were fed into a low-impedance Medusa Biological Amplifier System (RA4LI, TDT), which delivered the signal to the RZ6 digital signal processing hardware. The recorded signals were filtered using a 0.5–1 kHz bandpass filter, and the ABR waveforms in response to 512 tone bursts were averaged. ABR thresholds for each frequency were determined using BioSigRZ software. Peak amplitudes (mV) and peak latencies (ms) were calculated from the waveform signal of the click-evoked ABRs as input/output (I/O) functions with an increasing stimulus level (20–90 dB SPL). Distortion product otoacoustic emission (DPOAE) was measured using a combination TDT microphone–speaker system. Primary stimulus tones were produced using an RZ6 digital signal processor with SigGenRZ software and delivered by a custom probe with an ER 10B+ microphone (Etymotic, Elk Grove Village, IL, USA) and MF1 speakers positioned in the ear canal. The primary tones were set at a frequency ratio (f2/f1) of 1.2 with target frequencies of 6, 8, 10, 12, 16, 18, 24, and 30 kHz. The f2 intensity levels were the same as the f1 intensity levels (L1 = L2). The sounds resulting from the primary tones were received by the ER 10B+ microphone and recorded using an RZ6 digital signal processor. The DPOAE I/O functions were determined at specific frequencies (6 and 30 kHz) with a frequency ratio (f2/f1) of 1.2 and equal intensity levels (L1 = L2). The intensity level of the primary tones was increased from 20 to 80 dB SPL in 5 dB SPL increments. Fast Fourier transform (FFT) was performed at each primary tone for the DP grams and each intensity for the I/O functions using BioSigRZ software to determine the average spectra of the two primaries, the 2f1–f2 distortion products, and the noise floors.

### Scanning electron microscopy (SEM)

Inner ear samples were prepared for SEM analysis as described previously^19^. Inner ears were dissected from 4-week-old *Myh1*^−*/*−^ mice and fixed with 2% paraformaldehyde containing 2.5% glutaraldehyde in 0.1 M sodium cacodylate buffer (pH 7.4) for 2 h at room temperature. After fixation, the cochlear epithelium and tectorial membrane were separated and fixed overnight at 4 °C with 2.5% glutaraldehyde in 0.1 M sodium cacodylate buffer (pH 7.4) containing 2 mM calcium chloride and 3.5% sucrose. The fixed samples were washed three times with 0.1 M sodium cacodylate buffer containing 2 mM calcium chloride for 20 min at 4 °C and then postfixed using an osmium tetroxide (OsO_4_)/thiocarbohydrazide (OTO) protocol. The samples were dehydrated using a graded ethanol series, dried using a critical point dryer (Leica EM CPD300, Wetzlar, Germany), fixed on a stub, and coated with platinum to a thickness of 20–30 nm using a sputter coater (ACE600; Leica Microsystems). The platinum-coated samples were mounted on a stub holder and imaged using a Schottky emission scanning electron microscope (JSM-IT500, JEOL, Tokyo, Japan).

### Electrophysiology

Voltage-clamp experiments in mouse OHCs or human embryonic kidney (HEK) 293T cells (American Type Culture Collection, USA; #CRL-3216) were performed in standard whole-cell configurations. All recordings were performed at room temperature (22–25 °C). Microglass pipettes (World Precision Instruments, USA) were fabricated using a PP-830 single-stage glass microelectrode puller (Narishige, Japan) with a resistance of 2–5 MΩ. The liquid junction potential was rectified using an offset circuit prior to each recording. Currents were recorded using an Axopatch 700B amplifier and Digidata 1550 A interface, digitized at 100 kHz, and lowpass filtered at 10 kHz using pClamp software 10.7 (Molecular Devices, USA). The whole-cell voltage-clamp configuration was verified by measuring the series resistance to less than 10 MΩ, which was not compensated before each recording.

In the whole-cell voltage-clamp configuration, a sine wave stimulus with a 10 mV amplitude and 1 kHz frequency was overlapped with a ramp pulse protocol from −150 mV to 100 mV for a 250 ms duration. The holding potential was −70 mV. For each cell, at least two recordings were made to ensure stable measurement conditions.

The calculation of nonlinear capacitance (NLC) was adopted from previous studies^17,20^. The admittance (*Y(ω)*) of the system was determined through a spectral analysis, whereas the DC conductance (*b*) was extracted from the steady-state current preceding the sine wave stimulus. The circuit parameters, including capacitance (*C*), membrane resistance (*R*_*m*_), and series resistance (*R*_*s*_), were calculated as follows:

$${C}_{{\rm{m}}}=\frac{1}{\omega B}\frac{{\left({A}^{2}+{B}^{2}-{Ab}\right)}^{2}}{{\left(A-b\right)}^{2}+{B}^{2}}$$

$$b=\frac{1}{{R}_{{\rm{in}}}}=\frac{{I}_{{\rm{in}}}}{{V}_{{\rm{in}}}}$$

$$Y=\frac{{\rm{FFT}}\left(I\right)}{\Delta V}$$

$$A=\mathrm{Re}\left(Y\right)$$

$$B={\rm{Im}}\left(Y\right)$$

FFT is the fast Fourier transform, and Re*(Y)* and Im*(Y)* are the real and imaginary components, respectively. The capacitance was fit with a derivative of the Boltzmann equation as follows:

$${C}_{{\rm{m}}}={C}_{{\rm{lin}}}+\frac{{Q}_{\max }}{\alpha {e}^{\frac{V-{V}_{h}}{\alpha }}{\left(1+{e}^{-\frac{V-{V}_{h}}{\alpha }}\right)}^{2}}$$

$$\alpha =\frac{{ze}}{{kT}}$$

where *V*_*h*_ is the maximal activation voltage, *Q*_max_ is the maximal charge transfer between the plasma membrane, *α* is the slope factor for the voltage-dependent charge transfer, *z* is the charge valence, *e* is the electron charge, *k* is Boltzmann’s constant, and *T* is the absolute temperature. An in-house Python script was used for the NLC calculation and analysis.

### Preparation of the organ of Corti for electrophysiology

Organs of Corti were acutely isolated from mice at 21–28 days after birth. The animals were anesthetized with isoflurane (Sigma-Aldrich) and euthanized via decapitation. The bone surrounding the apical turn of the cochlea was removed, and the apical turn of the organ of Corti was carefully detached using forceps and subsequently separated from the lateral cochlear wall, stria vascularis, modiolus, and tectorial membrane. The whole dissection procedure was performed in a standard extracellular solution containing 144 mM NaCl, 5.8 mM KCl, 10 mM HEPES, 5.6 mM glucose, 0.7 mM NaH_2_PO_4_, 1.3 mM CaCl_2_, 0.9 mM MgCl_2_, and 10 mM sorbitol, adjusted to pH 7.4 using NaOH. The cochlea was immobilized onto a 12 mm diameter coverslip with a stainless steel pin and Sylgard and mounted on an upright microscope ECLIPSE FN1 (Nikon, Japan).

### Solutions

The whole-cell voltage-clamp experiment was conducted using a standard extracellular bath solution containing 144 mM NaCl, 5.8 mM KCl, 10 mM HEPES, 5.6 mM glucose, 0.7 mM NaH_2_PO_4_, 1.3 mM CaCl_2_, 0.9 mM MgCl_2_, and 10 mM sorbitol, adjusted to pH 7.4 with NaOH. The standard pipette solution used to record electromotility contained 140 mM CsCl, 10 mM HEPES, 1 mM EGTA, 3 mM Mg-ATP, and 1 mM MgCl_2_ and was adjusted to pH 7.2 with KOH.

### Immunofluorescence staining and whole-mount assay

The cochleae were isolated from *Myh1*^*+/+*^, *Myh1*^+*/*−^, or *Myh1*^−*/*−^ C57BL/6 mice at postnatal Day 30, fixed with 4% paraformaldehyde overnight, washed with phosphate-buffered saline (PBS), decalcified with 10% ethylenediaminetetraacetic acid (pH 7.2) for 7 days, transferred to a series of gradient ethanol solutions, immersed in dimethylbenzene, and finally embedded in paraffin for sectioning. For immunostaining, the sections were deparaffinized in xylene and rehydrated in gradient ethanol solutions. The cells were incubated in blocking buffer containing 10% donkey serum and 1% bovine serum albumin for 1 h at room temperature. The samples were incubated overnight at 4 °C with primary antibodies diluted in blocking buffer. After being washed with PBS, the samples were incubated with secondary antibodies and 4′,6-diamidino-2-phenylindole (DAPI) for 30 min at room temperature, washed, and covered with mounting medium and cover slips. Images were obtained with an LSM 780 microscope (Carl Zeiss). Anti-MYH1 (LSbio, LS-C346145), anti-MYO7A (Santa Cruz Biotechnology, sc-74516), and anti-Prestin (Santa Cruz Biotechnology, sc-293212) antibodies were obtained from commercial sources. Alexa Fluor 488- and Alexa Fluor 594-conjugated secondary antibodies and DAPI were obtained from Invitrogen.

### OHC circuit modeling

We assumed that three channels are responsible for major ionic conductance in OHCs: a mechanoelectrical transducer (MET) channel, a small conductance K^+^ channel, and a large conductance K^+^ channel, *I*_*K,n*_, whose molecular identity is KCNQ4.

The closed OHC circuit can be expressed using the following equation:

$${C}_{m}\frac{d{V}_{m}}{{dt}}+{I}_{\rm{MET}}+{I}_{K,s}+{I}_{K,n}=0$$

We implemented the NLC of the OHC for the membrane capacitance as follows:

$${C}_{m}={C}_{{lin}}+\frac{{Q}_{\max }}{\alpha {e}^{\frac{V-{V}_{h,{Pres}}}{\alpha }}{\left(1+{e}^{-\frac{V-{V}_{h,{Pres}}}{\alpha }}\right)}^{2}}$$

The gating of the MET channel was as follows^21,22^:

$${n}_{\rm{MET},\infty }=\frac{1}{1+{e}^{-\frac{\mu -{x}_{0}}{{s}_{1}}}\left(1+{e}^{-\frac{\mu -{x}_{0}}{{s}_{0}}}\right)}$$

$${n}_{\rm{MET}}+{\tau }_{\rm{MET}}\frac{d{n}_{\rm{MET}}}{{dt}}={n}_{\rm{MET},\infty }$$

$${g}_{\rm{MET}}\left(\mu \right)=n{G}_{\rm{MET}}$$

$${I}_{\rm{MET}}={g}_{\rm{MET}}\left(\mu \right)\left({V}_{m}-{EP}\right)$$

The gating of the *K*_*s*_ and *K*_*n*_ channels was as follows^23^:

$${n}_{K,s/n,\infty }=\frac{1}{1+{e}^{-\frac{{V}_{m}-{V}_{h,K,s/n}}{{s}_{K,s/n}}}}$$

$${n}_{K,s/n}+{\tau }_{K,s/n}\frac{d{n}_{K,s/n}}{{dt}}={n}_{K,s/n,\infty }$$

$${g}_{K,s/n}={n}_{K,s/n}{G}_{K,s/n}$$

$${\tau }_{K,s}=12.3+0.5{e}^{-\frac{{V}_{m}}{39.9}}$$

$${\tau }_{K,n}=7.5\times \left(\frac{1}{1+{e}^{-\frac{{V}_{m}+92}{13.6}}}\right){e}^{-\frac{V}{28.2}}$$

$${I}_{K,s/n}={g}_{K,s/n}\left({V}_{m}-{E}_{K}\right)$$

The power of electromotility was calculated using the following equation:

$$\Delta P=\frac{1}{2}{\Delta {{C}_{m}\Delta V}_{m}}^{2}$$

The differential equation was solved by an in-house Python script using the NumPy and SciPy modules^24,25^.

### Research subjects

This study was approved by the Institutional Review Board of Severance Hospital, Yonsei University Health System (IRB #4-2015-0659). We obtained written informed consent from individuals with hearing loss for their participation in this study and for the publication of their clinical data. The participants were enrolled in the Yonsei University Hearing Loss (YUHL) cohort based on the following inclusion criterion: hearing loss without congenital CMV infection or other medical diseases primarily affecting hearing function. We prescreened for pathogenic biallelic variants in *SLC26A4* and *GJB2* or copy number variations (CNVs) in *STRC*, which are the most common genetic causes in East Asians^26,27^. In total, probands from 437 families with no pathogenic variants in these three known genes were enrolled in the YUHL cohort for exome sequencing, and the majority had NSHL. Among those 437 families, 151 (151/437, 34.5%) presented either pathogenic variants or CNVs within deafness-causing genes. Otherwise, the exome sequencing analysis of 286 families revealed no other pathogenic variants in known hearing loss genes.

### Subject evaluation

Pure tone and speech audiograms were obtained for all patients and their affected and unaffected family members. Pure tone air (250–8000 Hz) and bone conduction (250–4000 Hz) thresholds were measured using a clinical audiometer in a double-walled audio booth. The degree of hearing loss was determined by averaging the air conduction thresholds at 500, 1000, 2000, and 4000 Hz. Steady-state auditory responses were also determined for young babies. Temporal bone computed tomography and magnetic resonance imaging were performed to evaluate inner ear abnormalities.

### Whole-exome sequencing (WES)

WES was performed as described previously. Briefly, genomic DNA was extracted from the peripheral blood or saliva samples of the affected individuals and their parents (where available) and subjected to exome capture using an Agilent SureSelect V5 enrichment capture kit (Agilent Technologies). The enriched library was then sequenced on an Illumina NovaSeq 6000 sequencing platform (151 bases, paired-end), and sequence reads were mapped to the human reference genome assembly (NCBI build 37/hg19) using CLC Genomic Workbench (version 9.5.3) software (Qiagen). After alignment, variants with a minimum coverage of two were subjected to a variant calling process using the “Basic Variant Caller” function in CLC and then annotated by Variant Effect Predictor. Single nucleotide polymorphisms reported in the dbSNP (version 147) database were excluded. Nonsynonymous variants or variants located within splice sites were then subjected to further genetic evaluation.

### Three-dimensional structural modeling

ColabFold and AlphaFold2 were used to construct the structures of the WT and mutant MYH1 proteins^28,29^. Due to the flexibility of the C-terminal region in MYH1 and the limited total length of the input sequences, we focused only on the head domain of MYH1, which corresponds to residues 1–843. The ColabFold program was downloaded from the website https://github.com/sokrypton/ColabFold and run locally. For protein modeling, the number of cycles was set to six, five models were predicted, and the top-ranked structure was selected for further analysis. The model type variable was AlphaFold2-multimer-v2. ChimeraX software was used for the three-dimensional visualization of the proteins^30^.

### Molecular dynamics simulations

The predicted structures of WT and mutant MYH1 were used as templates for molecular dynamics (MD) simulations. The system was initially neutralized and solvated in a 150 mM KCl environment using a CHARMM-GUI web server^31,32^. The final system was contained in an ~120 × 120 × 240 Å^3^ box, with ~105,000 water molecules and a total of ~330,000 atoms. All the simulations were performed with the CHARMM36m force field and GROMACS software^33,34^.

The particle mesh Ewald algorithm was used to evaluate the long-distance electrostatic interactions. The van der Waals interactions for a smoothing function were between 10 and 12 Å. The system was minimized for 5000 steps using the steepest descent algorithm. Then, 125 ps equilibration steps were applied to the protein, and the restraints were gradually reduced to zero. A complete electronic evaluation was performed every 2 fs using the SHAKE algorithm^35^. The pressure and temperature were, respectively, maintained at 1 atm and 298 K throughout the simulation using the Nosé–Hoover Langevin piston method and Langevin dynamics, respectively^36,37^. No anisotropic cell fluctuations were observed. For each system, a 100 ns simulation was performed, and snapshots were saved at 100 ps intervals for further analysis.

The root mean square deviation (RMSD) and root mean square fluctuation (RMSF) were calculated using Visual Molecular Dynamics (VMD) software^38^. For principal component analysis (PCA), protein trajectories were extracted every 100 ps and calculated using Bio3D software in R^39,40^.

### Surface biotinylation and immunoprecipitation

The experiments were performed as described previously^41,42^. Anti-Myc (sc-40) and anti-aldolase A1 (sc-12059; Santa Cruz Biotechnology, Dallas, TX, USA) antibodies were purchased from commercial sources. Surface biotinylation was performed with 0.3 mg/mL EZ-Link Sulfo-NHS-SS-Biotin and streptavidin (Thermo Fisher Scientific). Immunoprecipitation and coimmunoprecipitation (co-IP) were performed using EZview Red Anti-FLAG M2 Affinity Gel (Sigma) and Pierce Anti-c-Myc Magnetic Beads (Thermo Fisher Scientific).

### Immunoblotting

Immunoblotting was performed using primary antibodies, including anti-MYH1 (LS-C346145, LSBio), anti-actin (sc-18262, Santa Cruz Biotechnology), anti-Myc (2276T, Cell Signaling Technology, Danvers, MA, USA) and anti-Flag (14793T, Cell Signaling Technology) antibodies at a 1:1000 dilution, followed by the corresponding anti-isotype secondary antibodies (Santa Cruz Biotechnology) at a 1:2000 dilution. The signals were visualized using the SuperSignal West Pico Kit (Thermo Fisher Scientific).

### Fourier transform traction microscopy (FTTM)

Traction force microscopy was performed as described previously^43–45^. Briefly, transfected/untransfected COS-7 cells were plated sparsely on a polyacrylamide (PAA) gel coated with fibronectin (Thermo Fisher Scientific) and allowed to adhere and stabilize for 24 h. Phase contrast and fluorescence images of microbeads (Molecular Probes, Eugene, OR, USA) embedded near the gel apical surface of each cell were captured, while a fluorescence image of the same region and focal plane in the gel was captured after cell detachment with trypsin (traction-free reference). The microbead displacement between the two images was measured by identifying the coordinates of the peak of the cross-correlation function^43,44^. The traction field was computed using the elastic property of the gel (Young’s modulus of 20 kPa and Poisson’s ratio of 0.48), and the displacement field was computed using a MATLAB code.

### Statistical analysis

All the quantitative data were obtained from at least three independent experiments. Significant differences between two groups were examined using Student’s *t*-tests or two-way analysis of variance (ANOVA) with Bonferroni correction for multiple comparisons using GraphPad Prism 7.0 software (GraphPad Software, San Diego, CA, USA). Two-sided *p* values of <0.05 were considered significant.

## Results

### *Myh1*^*−/−*^ mice exhibit an impaired hearing phenotype due to outer hair cell dysfunction

**Fig. 1:**
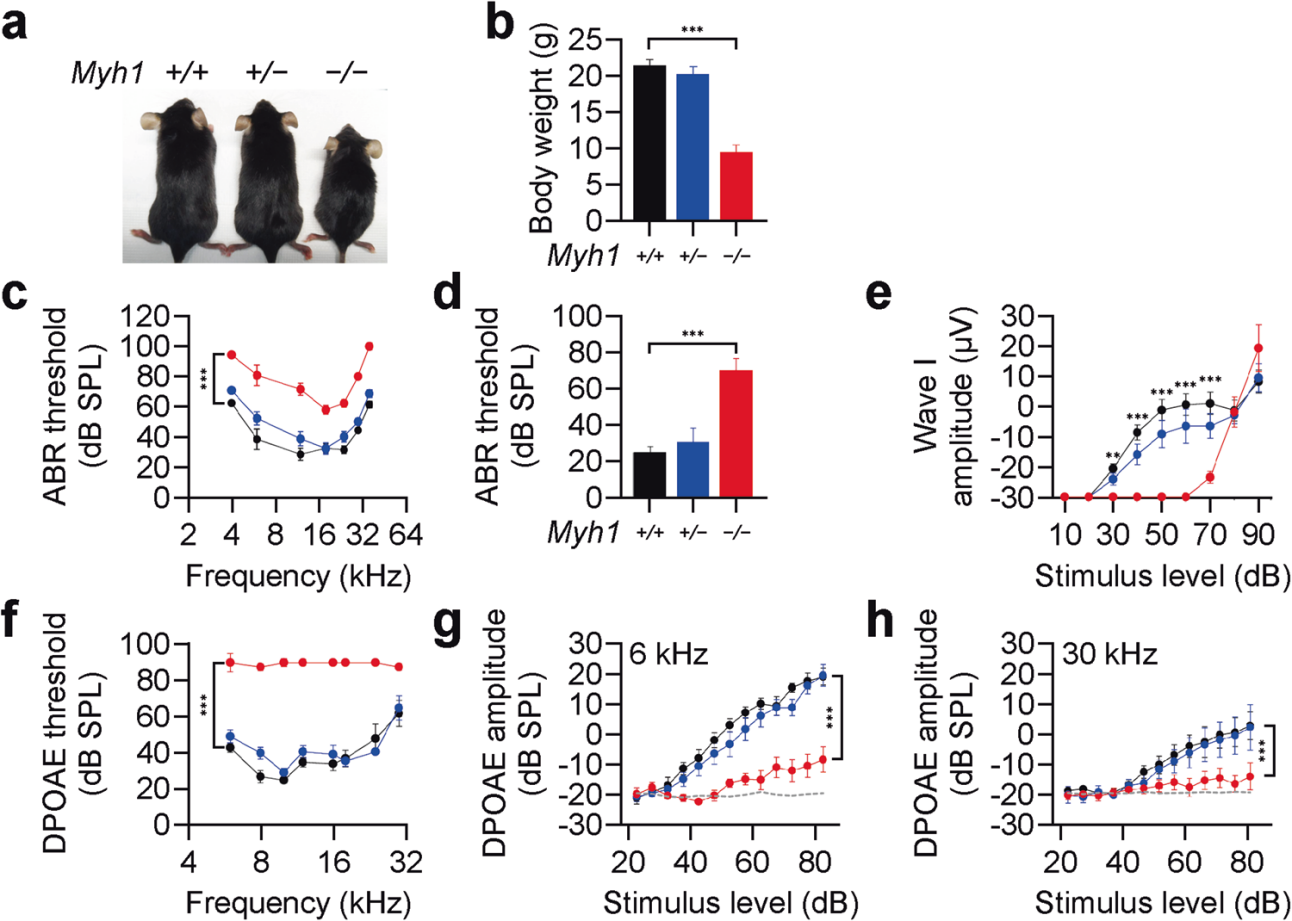
Hearing loss in *Myh1*^*−/−*^ mice due to outer hair cell dysfunction. **a** Overall appearance of *Myh1*^*+/+*^, *Myh1*^*+/−*^, and *Myh1*^*−/−*^ mice at postnatal Day 30. **b** Body weights of the *Myh1*^*+/+*^, *Myh1*^*+/−*^, and *Myh1*^*−/−*^ mice. **c** Tone-pip ABR thresholds, **d** click ABR thresholds, and **e** ABR I/O functions of the *Myh1*^*+/+*^, *Myh1*^*+/−*^, and *Myh1*^*−/−*^ mice at postnatal Day 30. **f** DPOAE thresholds and **g** DPOAE I/O functions at 6 kHz and **h** 30 kHz**. b**–**h** *Myh1*^+/+^ (black, *n* = 5), *Myh1*^+/−^ (blue, *n* = 7), and *Myh1*^−*/*−^ (red, *n* = 7) mice. The data are presented as the means ± SEMs. Statistical comparisons were performed using two-way ANOVA with Bonferroni correction for multiple comparisons in (**b**–**h**). **P < 0.01 and ***P < 0.001.

We generated *Myh1*^−*/*−^ mice (Fig. 1a) to understand the role of MYH1 in the inner ear; the mice were viable and fertile but smaller in size with a significantly lower body weight than *Myh1*^*+/−*^ and *Myh1*^*+/+*^ mice at postnatal Day 30 (Fig. 1b). We determined the hearing sensitivity of *Myh1*^−*/*−^ mice by measuring the ABR threshold in response to click stimuli of mixed broadband or pure tone sounds at individual frequencies on postnatal Day 30 (Fig. 1c, d). The thresholds for both click and pure tone stimuli at all frequencies were significantly increased in the *Myh1*^−*/*−^, but not in the *Myh1*^*+/*−^ mice, compared with those in the *Myh1*^*+/+*^ mice (Fig. 1d). ABR wave I amplitudes, which reflect the summed responses of auditory nerve fibers, were significantly lower in response to click stimuli up to 70 dB in the *Myh1*^−*/*−^ mice than in the *Myh1*^*+/+*^ or *Myh1*^*+/*−^ mice (Fig. 1e). However, the ABR wave I amplitudes evoked by higher-intensity stimuli (>80 dB) did not differ significantly among any of the genotypes. These results suggest that the machinery by which inner hair cells (IHCs) stimulate spiral ganglion neurons is intact and that the reduced hearing sensitivity indicated by elevated ABR thresholds is likely due to OHCs failing to act as cochlear amplifiers. We tested this hypothesis by analyzing DPOAE thresholds, which reflect the cochlear amplification function of OHCs. Compared with *Myh1*^*+/+*^ or *Myh1*^*+/−*^ mice, *Myh1*^−*/*−^ mice presented significantly higher DPOAE thresholds at all frequencies (Fig. 1f). In addition, DPOAE amplitudes were significantly reduced at both low (6 kHz) and high (30 kHz) frequencies in *Myh1*^−*/*−^ mice (Fig. 1g, h), suggesting that OHC dysfunction is the main cause of hearing loss in *Myh1*^−*/*−^ mice. SEM revealed that the stereociliary bundles of OHCs in 4-week-old *Myh1*^−*/*−^ mice were comparable to those in *Myh1*^*+/*−^ mice (Supplementary Fig. 1). Consistently, the imprints on the undersurface of the tectorial membrane, which represent connections between the tallest stereocilia and tectorial membrane, also appeared normal in the *Myh1*^−*/*−^ mice (Supplementary Fig. 1). These results suggest that *Myh1* is required for normal hearing, most likely by contributing to OHC function rather than stereociliary morphology. Furthermore, these findings confirm that *MYH1* is linked to hearing loss with autosomal recessive inheritance in both humans and mice.

### Myh1 is expressed in multiple cell types in the cochlea

**Fig. 2:**
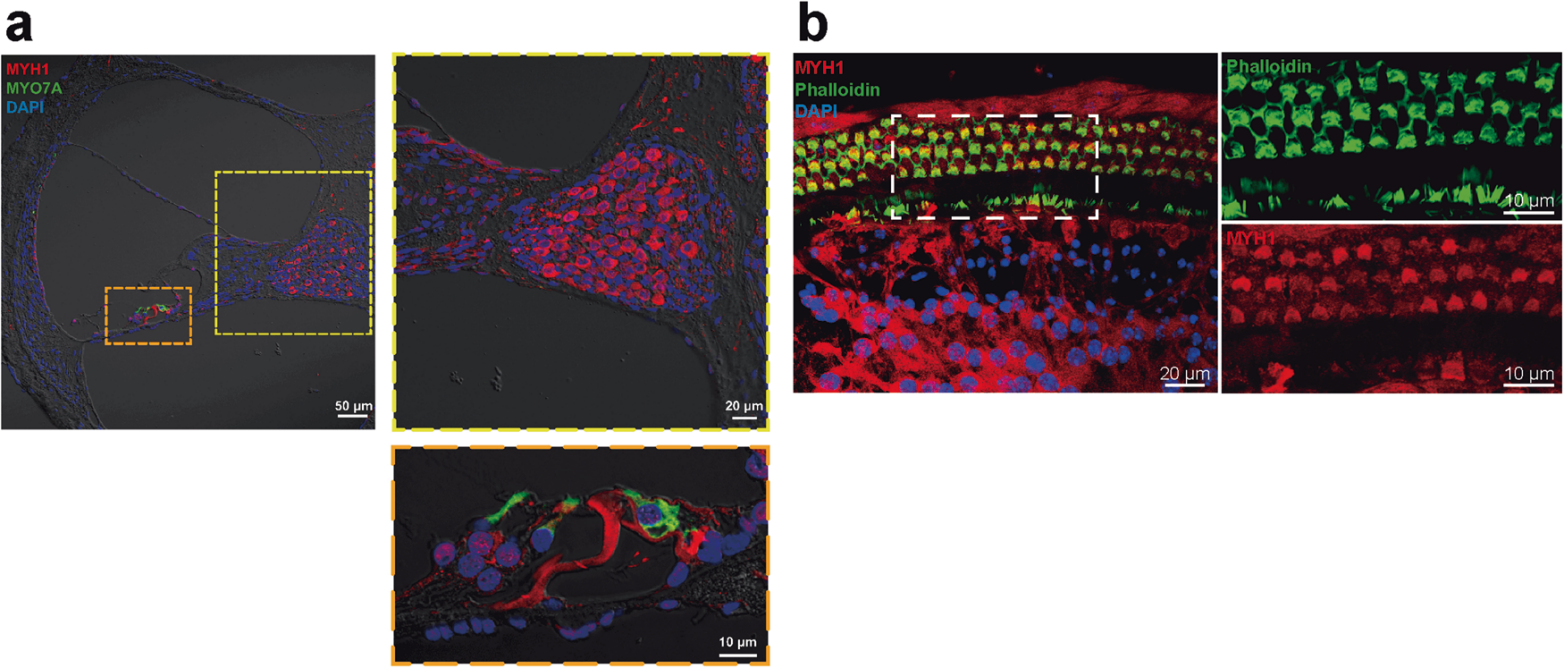
Myh1 expression in the organ of Corti. **a** Cross section of the inner ear from C57BL6/N mice at postnatal Day 30. MYH1, red; MYO7A, green. **b** Whole-mount images of the inner ears of C57BL6/N mice at postnatal Day 30. MYH1, red; phalloidin, green.

In mammals, at least ten different myosin heavy chain isoforms, which are encoded by different genes of the MYH family, have been described in striated, smooth, and nonmuscle cells^11^. Given that the expression of Myh1 has not been described in the inner ear, we performed immunofluorescence staining and a whole-mount assay using cochleae from mice at postnatal Day 30. Myh1 was detected in multiple cochlear structures and strongly expressed in spiral ganglion neurons with axonal sprouts and supporting cells around hair cells (Fig. 2a). Whole-mount imaging of the organ of Corti revealed that Myh1 was expressed not only in supporting cells but also in inner and OHCs (Fig. 2b).

### Prestin activity is lower in outer hair cells from *Myh1*^*−/−*^ mice

**Fig. 3:**
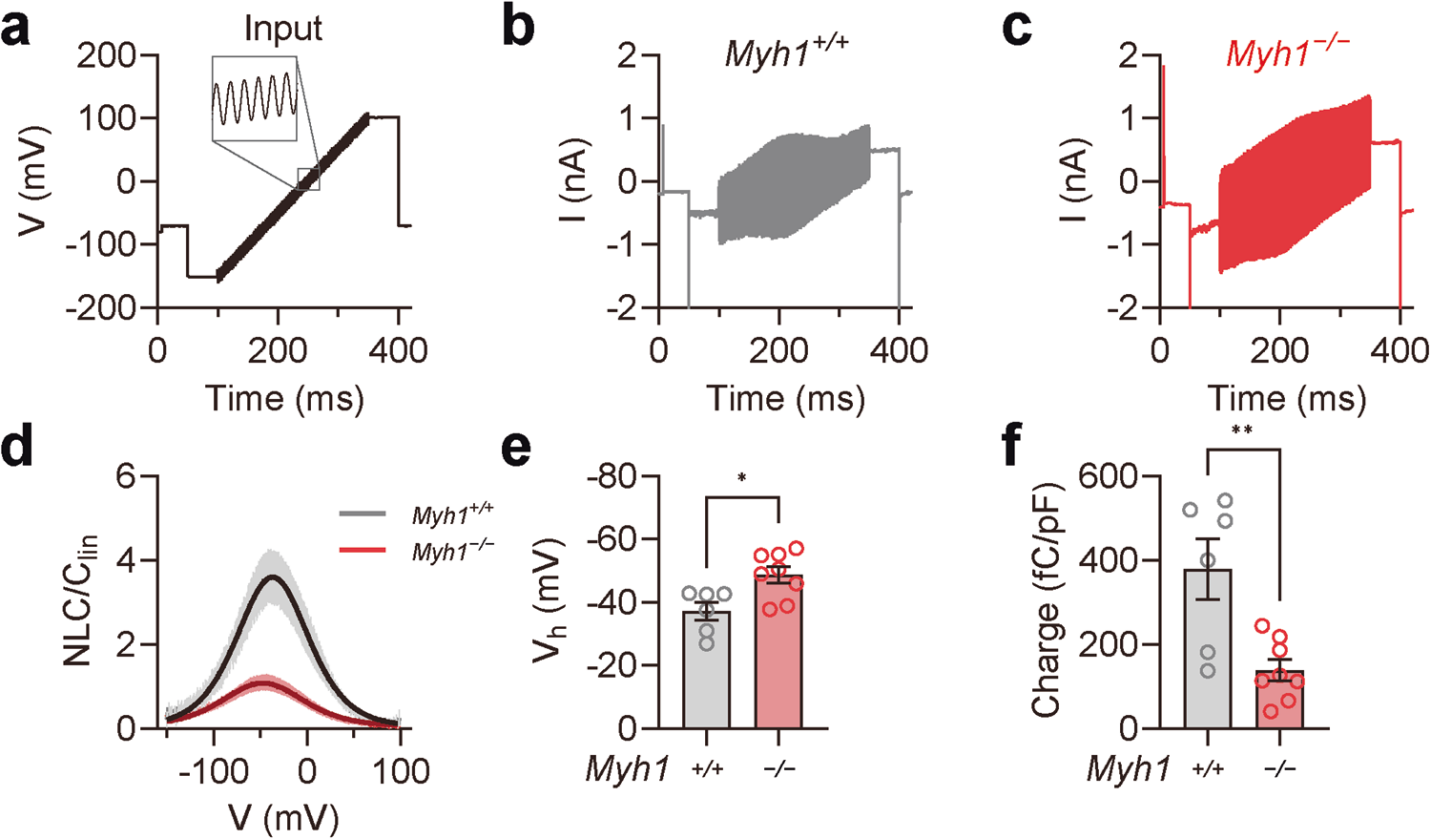
Impaired prestin activity in *Myh1*^*−/−*^ mouse outer hair cells. **a** Input voltage-clamp pulse protocol used for stimulating prestin in the outer hair cells. A 1 kHz sine wave with a 10 mV amplitude was simultaneously applied at −150 to 100 mV for a 250 ms duration. **b**, **c** Representative traces of *Myh1*^*+/+*^ and *Myh1*^*−/−*^ mouse outer hair cell recordings at postnatal Day 21. **d** Voltage-normalized nonlinear capacitance (NLC) relationship of *Myh1*^*+/+*^ (*n* = 6) and *Myh1*^*−/−*^ (*n* = 8) mouse outer hair cells. The bell-shaped curve was fit to the derivative of the Boltzmann equation. **e** *V*_*h*_ values calculated from the outer hair cells of *Myh1*^*+/+*^ and *Myh1*^*−/−*^ mice. **f** Normalized charge transfer density calculated from the outer hair cells of *Myh1*^*+/+*^ and *Myh1*^*−/−*^ mice. Data are presented as the means ± SEMs. Statistical comparisons were performed using two-sample independent *t*-tests in (**e**) and (**f**). *P < 0.05 and **P < 0.01.

Prestin, encoded by the *SLC26A5* gene, is a motor protein responsible for OHC electromotility and serves as the molecular basis of cochlear amplification^12,13^. We hypothesized that MYH1 may regulate prestin activity based on several lines of evidence: (i) the elevated DPOAE threshold in *Myh1*^*−/−*^ mice with intact tip links (Fig. 1 and Supplementary Fig. 1), indicating aberrant OHC electromotility; (ii) the direct interaction of MYH1 with the actin cytoskeleton; and (iii) the importance of the actin organization and distribution for electromotility in OHCs^14,46^. We thus examined prestin activity in the OHCs of postnatal Day 21 *Myh1*^*+/+*^ and *Myh1*^*−/−*^ mice using whole-cell voltage-clamp recordings. Surprisingly, when stimulated with the sine wave ramp pulse protocol (Fig. 3a), the current amplitude shift was greatly compromised in the OHCs of the *Myh1*^*−/−*^ mice (Fig. 3b, c). We then calculated the NLC from the current traces using the FFT method, as the NLC component is considered a direct measurement of prestin activity. Indeed, normalized NLC activity was impaired in *Myh1*^*−/−*^ mice, which was ~1/3 that of wild-type mice (Fig. 3d). Fitting the normalized NLC to the derivative of the Boltzmann equation allowed us to compare the maximal activation voltage (*V*_*h*_) and maximal charge transfer; *V*_*h*_ was significantly elevated, whereas the charge transfer was significantly decreased (Fig. 3e, f).

### Myh1 accounts for the majority of the electromotility power

**Fig. 4:**
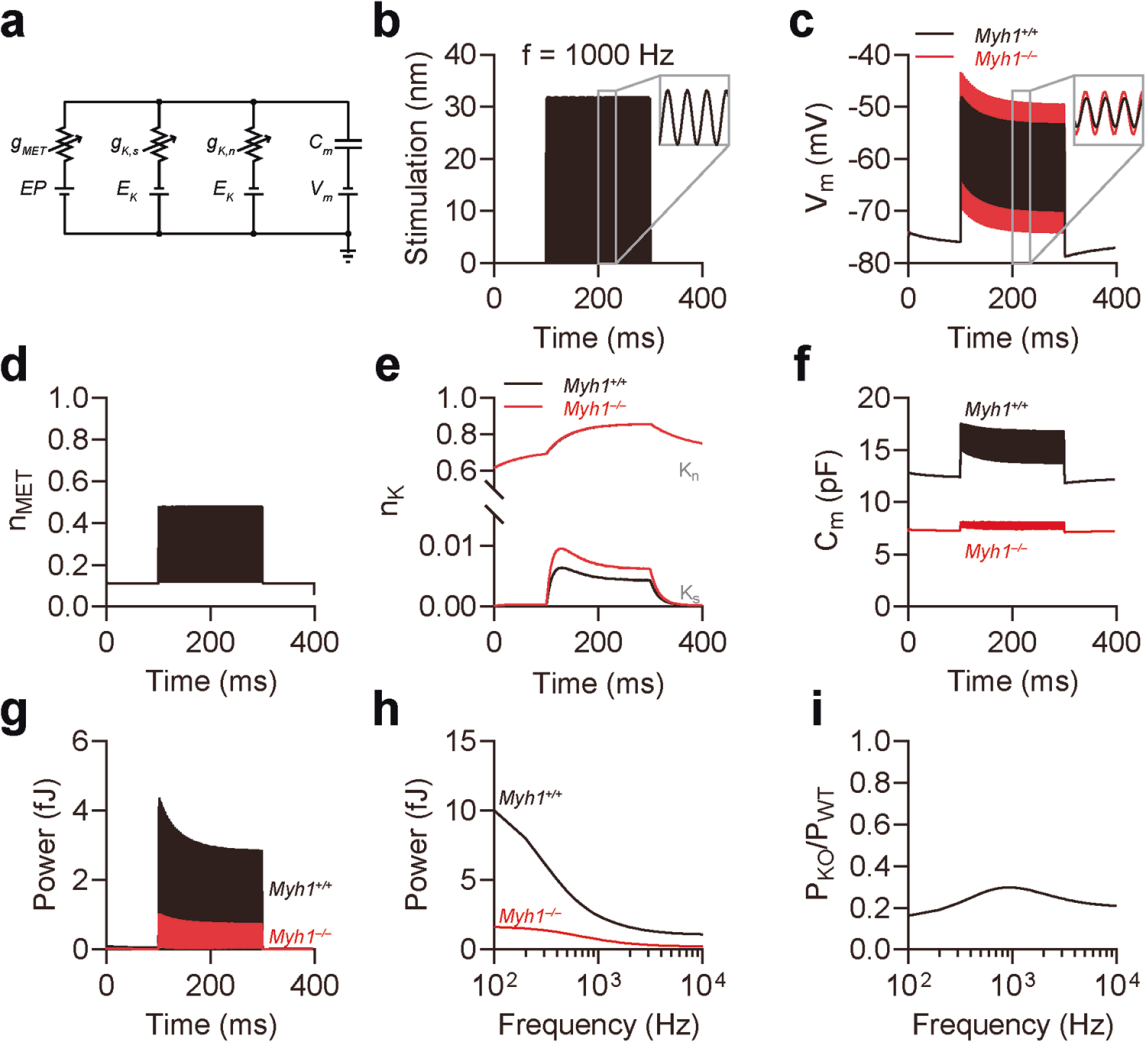
Simulation of *Myh1*^*+/+*^ and *Myh1*^*−/−*^ outer hair cells in response to sound stimulus. **a** Illustration of the outer hair cell (OHC) circuit. *g*_MET_ conductance of the mechanoelectrical transducer (MET) channel, *g*_*K,s*_ conductance of the small conductance K^+^ channel, *g*_*K,n*_ conductance of *I*_*K*,*n*_, corresponding to the KCNQ4 channel, *C*_*m*_ cell membrane capacitance, *EP* endocochlear potential, *E*_*K*_ K^+^ reversal potential, *V*_*m*_ basolateral membrane potential. **b** Stimulation of stereocilia at 1000 Hz for 200 ms. **c** Membrane potential oscillation by 1000 Hz stereocilia stimulation. **d** MET channel gating induced by 1000 Hz stereocilia stimulation. **e** K^+^ channel gating induced by 1000 Hz stereocilia stimulation. **f** Membrane capacitance oscillation induced by 1000 Hz stereocilia stimulation. **g** OHC electromotility power at 1000 Hz stereocilia stimulation. **h** OHC electromotility power across different frequencies of stereocilia stimulation. **i** Normalized electromotility power of *Myh1*^*−/−*^ OHCs compared to *Myh1*^*+/+*^ OHCs across different frequencies. In (**b–h**), *Myh1*^*+/+*^ OHCs are indicated by black lines, and *Myh1*^*−/−*^ OHCs are indicated by red lines.

We next aimed to determine the extent of the decrease in electromotility power caused by impaired prestin activity in OHCs from *Myh1*^*−/−*^ mice. For this purpose, we constructed a model of the OHC circuit based on previous studies^21–23^ (Supplementary Table 4). We hypothesized that three ion channels are responsible for the majority of ion conduction in OHCs, namely, the MET channel, small conductance voltage-activated K^+^ channel, and large conductance voltage-activated K^+^ channel, which corresponds to *I*_*K*,*n*_ and is mediated by KCNQ4^47,48^ (Fig. 4a). We also implemented NLC caused by prestin activity based on our electrophysiology experiments (Fig. 3). When the MET channel was stimulated with a 1000 Hz wave of 32 nm in amplitude (Fig. 4b), we observed fluctuations in the membrane potential with the corresponding frequency (Fig. 4c). Interestingly, although the resting membrane potential was near −73 mV, which was similar to the reported value^49^, the fluctuation in the membrane potential was greater in *Myh1*^*−/−*^ OHCs, implying that the *Myh1*^*−/−*^ OHC plasma membrane was less capable of storing electric charges. The gating of MET and *K*_*n*_ channels was similar between *Myh1*^*+/+*^ and *Myh1*^*−/−*^ OHCs (Fig. 4d, e), whereas *K*_*s*_ channel gating was greater in *Myh1*^*−/−*^ OHCs. Surprisingly, the membrane capacitance of *Myh1*^*−/−*^ OHCs was significantly lower in both the resting and fluctuating states (Fig. 4f). We next calculated the power generated by the capacitance of *Myh1*^*+/+*^ and *Myh1*^*−/−*^ OHCs, which was significantly lower in *Myh1*^*−/−*^ OHCs than in *Myh1*^*+/+*^ OHCs during the stimulation timeline (Fig. 4g). We next expanded our calculations across different stimulation frequencies from 100 to 10,000 Hz and observed that *Myh1*^*−/−*^ OHCs presented decreased electromotility power across all frequencies (Fig. 4h). When normalized to *Myh1*^*+/+*^ OHCs, *Myh1*^*−/−*^ OHCs had 16.2% and 29.8% electromotility power at 100 and 1000 Hz, respectively (Fig. 4i).

### *MYH1* variants identified from patients with hearing loss

We performed WES of a cohort of 437 families with hearing loss. A total of 151 of 437 families (34.6%) were molecularly diagnosed with either single nucleotide variants and insertions and deletions or CNVs in genes linked to nonsyndromic HL (https://hereditaryhearingloss.org/) or syndromic HL (https://www.omim.org/) (Supplementary Fig. 2a)^50–56^. Pathogenic variants in any of the previously reported genes were not detected in 286 families. After applying the variant filtering criteria (Supplementary Information; Materials and Methods, Supplementary Fig. 3, and Supplementary Table 1), we identified eight different biallelic variants in *MYH1* (myosin heavy chain 1; MIM: 16073) in five probands who were diagnosed with a recessive form of sensorineural hearing loss. The coverages of the targeted regions in the exome data for the 15-fold read depth were 94.4% (YUHL100-21), 96.9% (YUHL105-21), 97.4% (YUHL110-21), 92.5% (YUHL162-21) and 98.0% (YUHL624-21), and the average read depths were 54×, 68×, 73×, 94×, and 57×, respectively. These individuals also had biallelic variants in other genes (Supplementary Table 2). The eight identified variants of *MYH1* were missense variants, which are rare in both gnomAD v2.1.1 (https://gnomad.broadinstitute.org/) and Korean Variant Archive 2 (KOVA2; approximately *n* = 5305 individuals), with minor allele frequencies <0.005^57^. Sanger sequencing confirmed that the variants cosegregated with hearing loss as an autosomal recessive trait in five families (Supplementary Fig. 4). *MYH1* variants, including c.582G > C (p.Gln194His) and c.2977A > G (p.Ile993Val), c.2231A > C (p.Lys744Thr) and c.5416C > A (p.Gln1806Lys), c.1379T > C (p.Ile460Thr) and c.2438 A > T (p.Glu813Val), were present in the compound heterozygous state in probands from the YUHL110, YUHL105, and YUHL100 families, respectively (Supplementary Figs. 2b and 4). In the YUHL100 family, the affected status was segregated into another family member (YUHL100-22) with a shared audiological phenotype (Supplementary Fig. 5). Two homozygous variants of *MYH1*, c.2495C > G (p.Pro832Arg) and c.4617A > T (p.Gln1539His), were identified and segregated in the YUHL162 and YUHL624 families (Supplementary Figs. 2b and 4). However, according to the ACMG guidelines^58^, the clinical significance of *MYH1* variants identified in the cohort remains uncertain and requires further evaluation of the patients’ phenotypes (Supplementary Table 1). According to the International Mouse Phenotyping Consortium (IMPC; https://www.mousephenotype.org/), *Myh1*-knockout mice also present decreased bone mineral density, abnormal vocalization, decreased grip strength, and decreased urine creatinine levels^9^.

Among the probands from the five unrelated families, YUHL110-21 was a 17-year-old female with moderate-to-severe sensorineural hearing loss (Supplementary Fig. 2c) that occurred in the first decade of her life. She did not have any cochlear anomalies (Supplementary Fig. 2d). In dual-energy X-ray absorptiometry, the *z* scores for the femur neck and total hip (−1.4 and −1.6, respectively) indicated osteopenia, according to the 2013 Pediatric ISCD Positions (Supplementary Table 2)^59^. Her urine creatinine level (45.6 mg/dL) was normal, indicating no muscle myopathy. YUHL105-21 was a 3-year-old male with congenital hearing loss, which was more severe in the left ear (Supplementary Fig. 2c). He had no perinatal medical problems and no syndromic features. He did not have any inner ear anomalies, but had severe-to-profound hearing loss in the more severe ear (Supplementary Fig. 2d). He underwent cochlear implantation in the left ear, and the performance was good, with an aided threshold of 20–30 dB HL. Although his muscular development was normal and his urine creatinine level was within the normal range (68.7 mg/dL), the *z* scores determined from dual-energy X-ray absorptiometry were −1.6 and −1.7 for the femoral neck and total hip, respectively, indicating osteopenia (Supplementary Table 3). YUHL162-21 was a 2-year-old male with bilateral severe-to-profound congenital hearing loss, which manifested more severely in the left ear (Supplementary Fig. 2c). With no other clinical manifestations, the patient presented with bilateral cochlear anomalies classified as incomplete partition type I (Supplementary Fig. 2d; red arrows). Nonetheless, the laminar layers in the cochlea were well preserved, and residual hearing was detected bilaterally (Supplementary Fig. 2d). The individual underwent cochlear implantation in the left ear, with a good outcome (as high as a score of 6 in the categorized auditory performance) 1 year after implantation. As YUHL162-21 was only 2 years old, the muscular phenotype and bone mineral density values needed to be determined at follow-up. YUHL100-21 was a 63-year-old male with bilateral moderate hearing loss of uncertain onset (Supplementary Fig. 2c). Given that he had poor pronunciation and speaking proficiency, hearing loss was presumed to have initiated during childhood. His word recognition scores were 68 and 56% for the right and left ears, respectively. He did not exhibit any other syndromic features. YUHL100-22 was a 75-year-old female with moderate-to-severe hearing loss (Supplementary Fig. 5) and poor speech intelligibility (word recognition scores of 20 and 60% for the right and left ears, respectively). In the simplex YUHL624 family, YUHL624-21 had profound bilateral congenital sensorineural hearing loss (Supplementary Fig. 2c). YUHL624-21 also had cochleovestibular anomalies such as incomplete partition type I. She underwent cochlear implantation surgery at the age of 8 months, in which a cerebrospinal fluid gusher was noted. The outcome after 6 months was successful, with a score of 5 for categorized auditory performance. Based on these clinical manifestations, myopathy may be present at the subclinical level, as decreased muscle tone may lead to a decreased bone mineral density; however, the severity of osteopathy in this patient was clinically uncertain.

### *MYH1* variants are structurally different from the wild-type protein

**Fig. 5:**
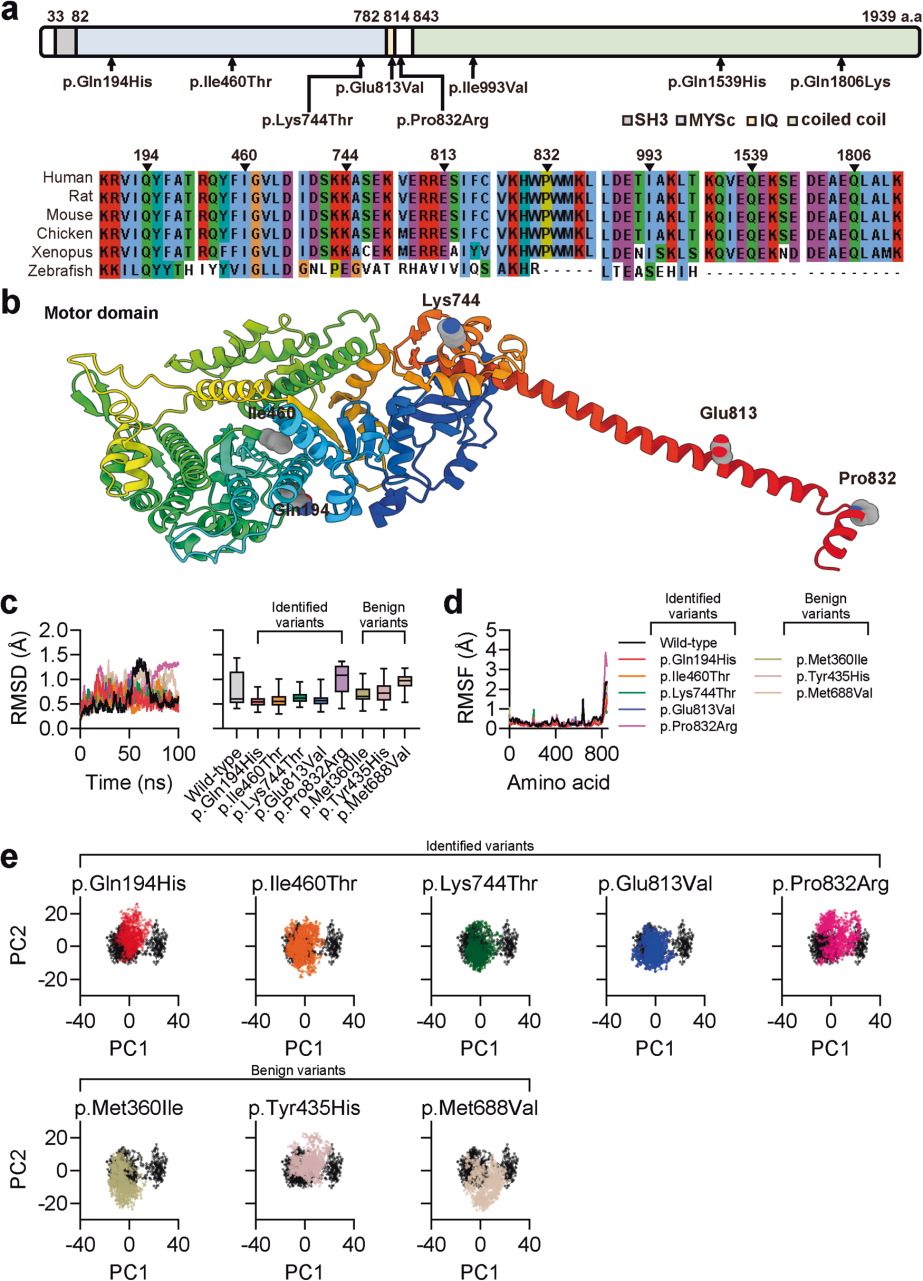
Molecular dynamics simulation of identified MYH1 variants. **a** Location of the *MYH1* variants in terms of the domain structure and amino acid conservation across other vertebrates. **b** The overall architecture of MYH1 (residues 1–843) predicted using AlphaFold2. The variant residues are indicated. **c** Root mean square deviation (RMSD) analysis of a 100 ns MD simulation of the wild-type and mutant MYH1 proteins. **d** Root mean square fluctuation (RMSF) analysis of the last 50 ns. **e** Principal component analysis (PCA) of MD simulation trajectories.

MYH1 is a myosin heavy chain that is predominantly expressed in adult skeletal muscle and contains an N-terminal globular head, SH3, and IQ domains, followed by a C-terminal coiled-coil rod-like tail^60^. Among the identified missense variants, three affected the motor domain, two affected the IQ domain, and the other three affected the tail domain of MYH1 (Fig. 5a). We questioned whether the *MYH1* variants identified in individuals with hearing loss were structurally different from the wild-type protein. No crystal structure is available for MYH1; therefore, ColabFold^28^ and AlphaFold2^29^ were used to predict the structure of the head domain of MYH1 corresponding to residues 1–843 (Fig. 5b). The overall prediction score was reasonably high for the majority of the residues, except for the N- and C-terminal loops, which were expected to be flexible (Supplementary Fig. 6). We then questioned whether *MYH1* variants were dynamically different from the wild-type protein, and thus modeled p.Gln194His, p.Ile460Thr, p.Lys744Thr, p.Glu813Val, and p.Pro832Arg variants with AlphaFold2^29^. Each protein was subsequently solvated, energetically minimized, and equilibrated for MD simulations. We included the benign variants p.Met360Ile, p.Tyr435His, and p.Met688Val as negative controls for the structural analysis, which were selected based on their allele frequency in the gnomAD database. During a 100 ns simulation, the root mean square deviation (RMSD) was lower in the last 50 ns for the p.Gln194His, p.Ile460Thr, p.Lys744Thr, and p.Glu813Val variants than for the wild-type protein or benign variants, indicating that these variants were structurally rigid (Fig. 5c). Notably, the p.Pro832Arg variant presented higher RMSD values in the last 50 ns of the simulation. A subsequent RMSF analysis of each amino acid revealed no major differences, except for the C-terminus of the p.Pro832Arg variant, which was consistent with its high RMSD and variant location (Fig. 5d). PCA of wild-type MYH1 revealed two major clusters, which were analyzed via the K-means clustering algorithm (Supplementary Fig. 7a). A comparison of these clusters highlighted the head domain loop and linker domain as the primary sources of structural differences (Supplementary Fig. 7b). Plotting the principal components of each variant against the wild-type protein revealed that the p.Gln194His, p.Ile460Thr, p.Lys744Thr, and p.Glu813Val variants clustered around a single cluster, indicating reduced flexibility of the linker domain (Fig. 5e).

### *MYH1* variants do not increase prestin activity compared to the wild-type protein

**Fig. 6:**
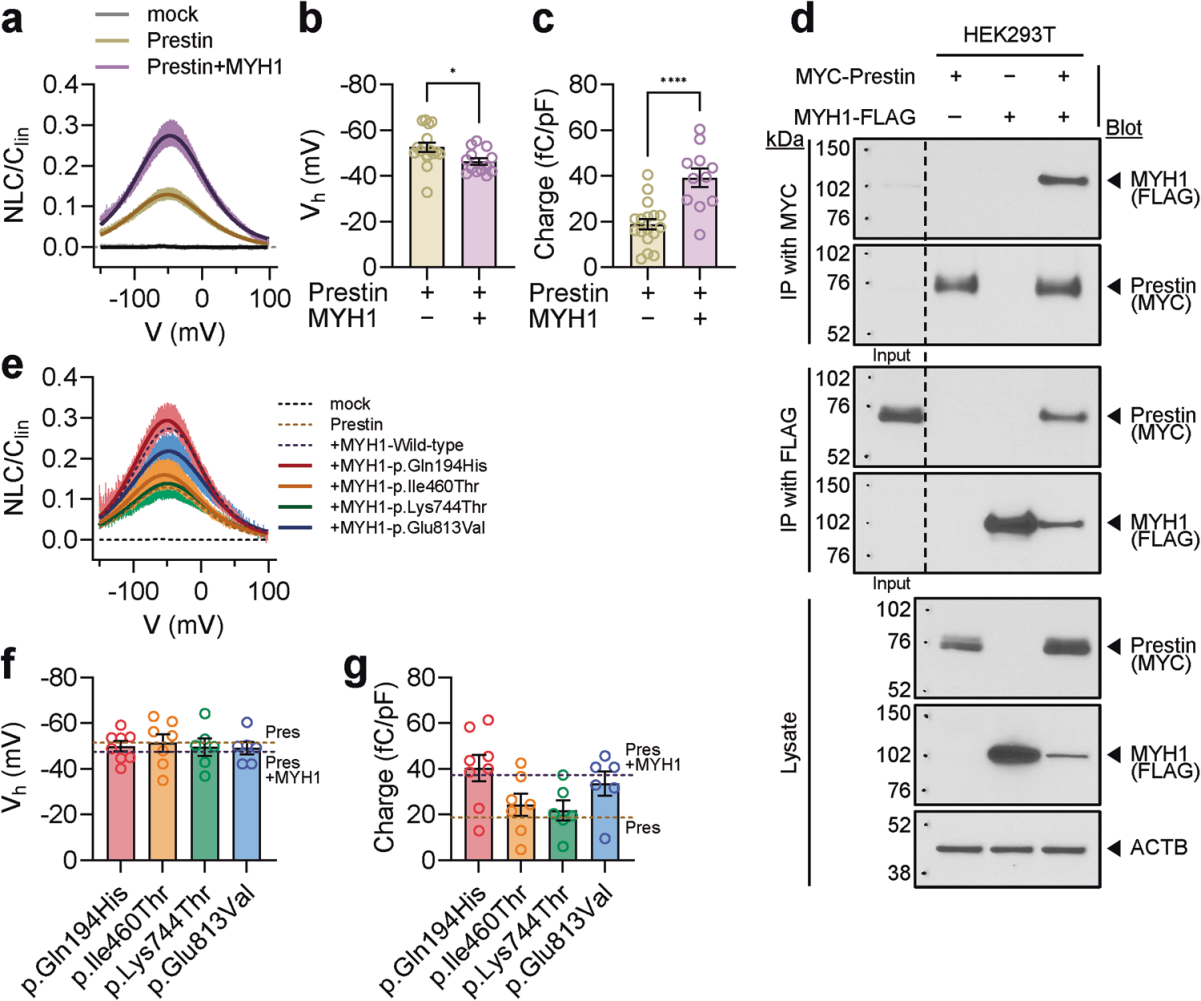
Prestin activity in wild-type or mutant MYH1-expressing cells. **a** Voltage-normalized nonlinear capacitance (NLC) relationship of overexpressed prestin with or without MYH1 in HEK293T cells. **b** *V*_*h*_ values measured in cells overexpressing prestin with or without MYH1. **c** Normalized charge transfer density measured in cells overexpressing prestin with or without MYH1. **d** Immunoprecipitation (IP) and coimmunoprecipitation (co-IP) of prestin- and MYH1-transfected HEK293T cells. The input lanes represent 1/10 of the immunoprecipitated cell lysate. **e** NLC relationship between overexpressed prestin and mutant MYH1. **f** *V*_*h*_ values and normalized charge transfer density measured from cells overexpressing prestin and mutant MYH1. **g** Normalized charge transfer density measured from cells overexpressing prestin and mutant MYH1. The data are presented as the means ± SEMs. Statistical comparisons were performed using two-sample independent *t*-tests in (**b**) and (**c**). *P < 0.05 and ****P < 0.0001.

Next, we investigated whether *MYH1* variants are also associated with impaired prestin activity. We expressed prestin with or without the MYH1 head domain in HEK293T cells. Unlike mock-transfected cells, cells expressing prestin presented robust signs of electromotility (Fig. 6a). Interestingly, prestin and MYH1 double-transfected cells exhibited greater electromotility than the prestin-only transfected cells (Fig. 6a). *V*_*h*_ was lower in the cotransfected cells, whereas the charge transfer was significantly higher (Fig. 6b, c). These results were consistent with the OHCs from the *Myh1*^*+/+*^ and *Myh1*^*−/−*^ mice (Fig. 3e, f). Next, we performed co-IP experiments in HEK293T cells to investigate the physical interaction between prestin and MYH1 (Fig. 6d). Interestingly, co-IP using both MYC and FLAG beads revealed a strong physical interaction between prestin and MYH1. While their direct interaction remains unclear, both prestin and MYH1 are likely regulated by actin cytoskeletal networks. We next transfected the mutant MYH1 with prestin and measured its electromotility. The p.Ile460Thr and p.Lys744Thr variants showed similar electromotility to the prestin-only transfected cells, whereas the p.Glu813Val variant exhibited moderately decreased electromotility compared with the wild-type MYH1 protein, and the electromotility of the p.Gln194His variant was similar to that of the wild-type protein (Fig. 6e). *V*_*h*_ and charge transfer also showed similar tendencies (Fig. 6f, g).

### MYH1 regulates the traction force of the cell membrane

**Fig. 7:**
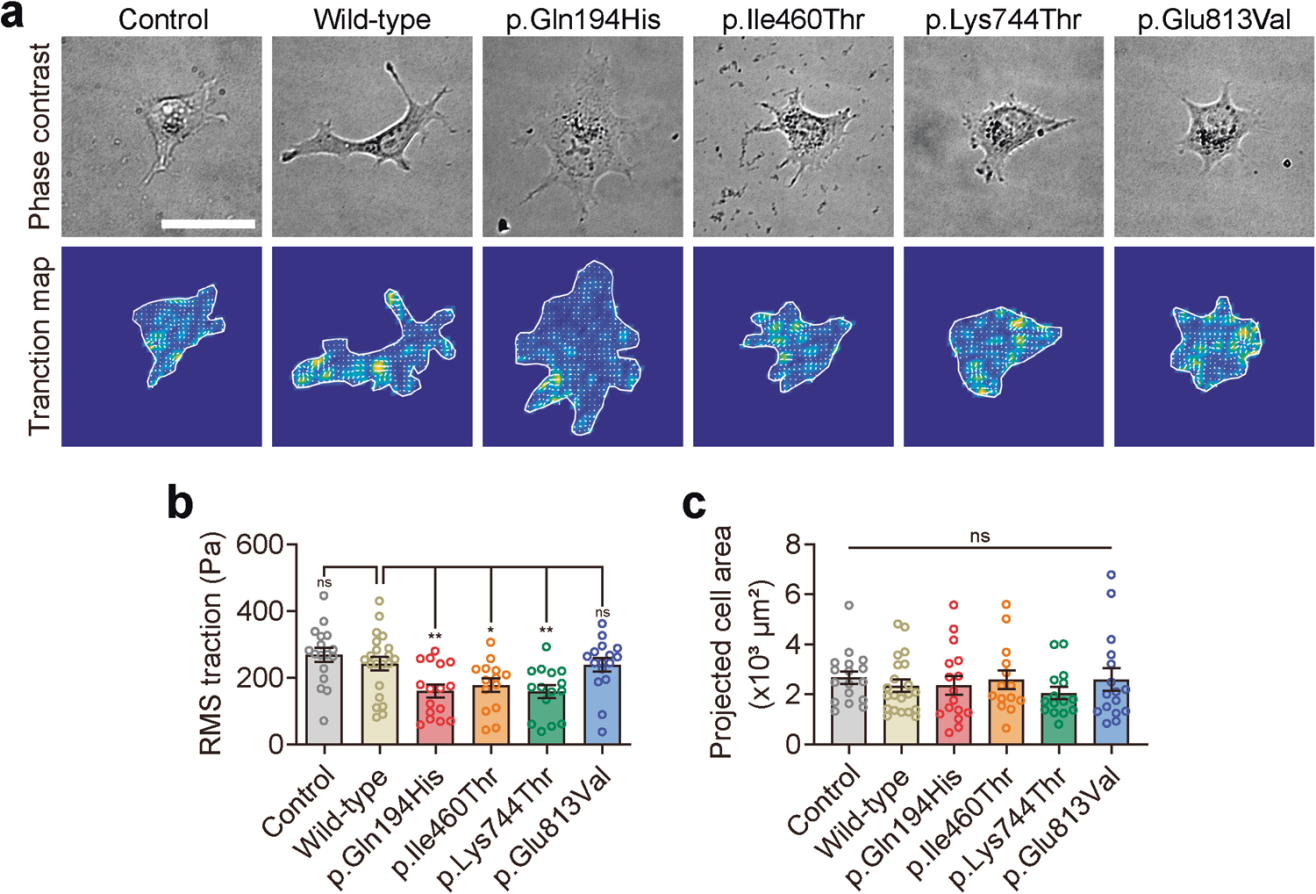
Traction stress maps of wild-type or mutant MYH1-expressing cells. **a** Representative phase contrast (top panel) and traction field images (bottom panel) of COS-7 cells adhered to an elastic polyacrylamide gel coated with fibronectin (Young’s modulus of 20 kPa with a Poisson’s ratio of 0.48). Scale bar = 50 µm. **b** Projected cell area measured in cells overexpressing wild-type or mutant MYH1. **c** Root mean square (RMS) traction measured in cells overexpressing wild-type or mutant MYH1. The data are presented as the means ± SEMs. Statistical comparisons were performed using two-way ANOVA with Bonferroni correction for multiple comparisons in (**b**) and (**c**). ns not significant; *P < 0.05; **P < 0.01.

We then investigated whether the increased prestin activity observed in the presence of MYH1 results from the direct modulation of prestin itself. However, the surface biotinylation of prestin was not affected by MYH1 in overexpressing HEK293T cells, and prestin localization in OHCs was similar between *Myh1*^*+/+*^ and *Myh1*^*−/−*^ mice (Supplementary Fig. 8). Thus, we hypothesized that MYH1 may affect the plasma membrane traction force, potentially exerting physical control over prestin activity. This hypothesis was derived from the observation that other factors that regulate membrane tension, such as cholesterol, have been shown to influence prestin activity^61,62^. We transfected COS-7 cells with wild-type MYH1 or its variants (p.Gln194His, p.Ile460Thr, p.Lys744Thr, and p.Glu813Val) to explore their mechanical effects. Wild-type MYH1 and its variants were observed throughout the COS-7 cell body, except for the nucleus (Supplementary Figs. 9 and 10), whereas other members of the myosin superfamily associated with hearing loss, such as *MYO3A* and *MYO15A*, have been shown to be localized to the tips of filopodia in COS-7 cells and stereocilia in hair cells^63,64^. We measured large-scale traction stress at the interface between adherent cells and the elastic matrix using Fourier transform force microscopy to assess how wild-type and variant MYH1 proteins function in cell mechanics (Fig. 7a). The wild-type and variant MYH1 proteins did not differ in terms of cellular expression, and the overexpression of MYH1 proteins in COS-7 cells induced cellular traction regardless of the variant type. However, the average traction stress generated differed between the wild-type and variant MYH1 proteins (Fig. 7b). Traction stress did not differ significantly between the cells expressing the wild-type and p.Glu813Val MYH1 proteins, whereas the cells expressing the other three MYH1 proteins (p.Gln194His, p.Ile460Thr, and p.Lys744Thr) presented a remarkable decrease in traction force (Fig. 7b). The cells transfected with the *MYH1* variants showed a similar level of spreading to the cells transfected with wild-type MYH1 when loaded onto a fibronectin-coated polyacrylamide gel with a Young’s modulus of ~20 kPa (Fig. 7c).

## Discussion

In this study, we successfully provided evidence for the function of Myh1 in murine hearing by performing functional studies in vivo. Consistent with previous publications^9^, *Myh1*^*−/−*^ mice presented impaired auditory function and noticeably elevated DPOAE thresholds across all frequencies. Our research revealed broad expression of MYH1 across multiple cochlear structures, including supporting cells and hair cells. We speculated that the hearing loss in *Myh1*^*−/−*^ mice might be due to OHC dysfunction and performed electrophysiology studies on electromotility to elucidate the role of MYH1 in OHCs. Through our analysis, we confirmed that reduced prestin activity was a major pathological characteristic in *Myh1*^*−/−*^ mice. Additionally, based on our simulations, MYH1 was responsible for at least 70% of the electromotility power generated in OHCs. These results establish an essential role for MYH1 in OHCs, which are responsible for cochlear amplification throughout the auditory system^65^.

Prestin is a motor protein responsible for generating electromotility in OHCs^12–14^. While in vitro expression of prestin successfully reproduces the electrophysiological properties of OHC electromotility, many extrinsic factors are involved in amplifying prestin activity. Examples include cholesterol, the actin–myosin complex, and temperature^46,61,66^. In addition to these factors, we propose that MYH1 also physically affects prestin activity by increasing the traction force of the cell membrane. Our experiments revealed that MYH1 interacts with prestin, resulting in several-fold higher prestin activity in OHCs than in the heterogenous expression system (Figs. 3 and 5). Although coexpression of MYH1 with prestin increased charge transfer and lowered *V*_*h*_, similar to the OHCs, the absolute NLC amplitude was still higher in the OHCs. We assume that unidentified molecular mechanisms specific to OHCs other than MYH1 do exist. These pathways could be due to the additional beta subunit, stiffening of the OHC membrane^14^, or prestin alignment for electromotility amplification^62^.

Here, we report five patients with autosomal recessive hearing loss in whom biallelic missense variants of *MYH1* were identified. According to the ACMG/AMP guidelines, the identified missense variants are of uncertain significance, and further genetic confirmation is required. Notably, three of the five patients also exhibited osteopenia, mirroring the reduced bone mineral density observed in *Myh1*-KO mice. Furthermore, variants of *MYH1* in the head domain caused decreases in prestin activity (p.Ile460Thr, p.Lys744Thr, and p.Glu813Val) and traction force (p.Gln194His, p.Ile460Thr, and p.Lys744Thr) in vitro compared with those of wild-type MYH1. These results suggest the hypomorphic nature and potential deleteriousness of the *MYH1* variants in the head domain.

The gnomAD database contains 197 *MYH1* alleles with loss-of-function variants, each having minor allele frequencies <0.005, suggesting the presence of potential disease-causing alleles^67^. In addition, compound heterozygous events of *MYH1* missense variants are expected to be rare. According to variant co-occurrence data in gnomAD, out of 125,748 individuals, 434 carried more than two *MYH1* variants with an MAF of <0.005. Among these 434 individuals, 43 were unphased, 390 carried *MYH1* variants in a *cis* configuration, and only 1 carried variants in a *trans* configuration. Compound heterozygous missense *MYH1* variants were reported as a likely pathogenic cause of childhood absence epilepsy in a child who also exhibited global developmental delays^68^. However, whether the child also has hearing loss is unclear. Data provided in gnomAD revealed two South Asian individuals homozygous for one of the *MYH1* variants (c.868C > T; p.Arg290Cys), suggesting that this variant is unlikely to cause a severe phenotype, such as global developmental delay.

Other members of the myosin heavy chain family are associated with hearing loss in humans. *MYH9* (MIM: 1607750) and *MYH14* (MIM: 608568), both of which are class II myosin heavy chains in nonmuscle cells, are implicated in autosomal dominant hearing loss, specifically DFNA17 (MIM: 603622) and DFNA4A (MIM: 600652), respectively^69,70^. In addition, unconventional myosins are implicated in hearing loss. Mutations in *MYO3A* (MIM: 606808) are responsible for DFNB30 (MIM: 607101)^64^, and *MYO6* (MIM: 600970) is mutated in DFNA22 (MIM: 606346) and DFNB37 (MIM: 607821)^71,72^. Mutations in *MYO7A* (MIM: 276903) are implicated in two forms of NSHL, DFNA11 (MIM: 601317) and DFNB2 (MIM: 600060)^73,74^. *MYO15A* (MIM: 602666) is also implicated in DFNB3 (MIM: 600316)^75^. Mutations in *MYO15A* are currently the third most common cause of hearing loss, with ~192 mutations identified to date. These mutations mostly cause prelingual severe-to-profound hearing loss, although some result in postlingual progressive hearing loss, likely depending on the nature of the mutation^76^. By combining our phenotype evaluation in mice with the IMPC database, we found that *Myh1*-knockout mice presented not only hearing impairment but also a decreased bone mineral density, abnormal vocalization, reduced grip strength, and lower urinary creatinine levels. While individuals with recessive *MYH1* variants did not consistently display all these phenotypes, three probands (YUHL624-21, YUHL110-21, and YUHL105-21) presented a decreased bone mineral density, which could be explained by a common myosin-related pathogenesis. Additional population-based studies are necessary to establish a strong genotype–phenotype correlation for *MYH1*.

In conclusion, our study elucidates the novel function of MYH1 in hearing. Its correlation with prestin activity implies that MYH1 plays an essential role in cochlear amplification and auditory functions in mice.

## Supplementary information

Supplemetary information

## Data Availability

All relevant data are included in the main manuscript and Supplementary Information. The Python script for simulating the OHC circuit is publicly available at https://github.com/doctroh/outer-hair-cell/tree/main.
