# Supplementary material for: MYH1 deficiency disrupts outer hair cell electromotility, resulting in hearing loss
Source: Exp Mol Med. 2024 Nov 1;56(11):2423–35. doi: 10.1038/s12276-024-01338-4 (PMC11612406; doi:10.1038/s12276-024-01338-4)
Supplement: Supplementary file 1 — Supplemetary information [file 12276_2024_1338_MOESM1_ESM.pdf]

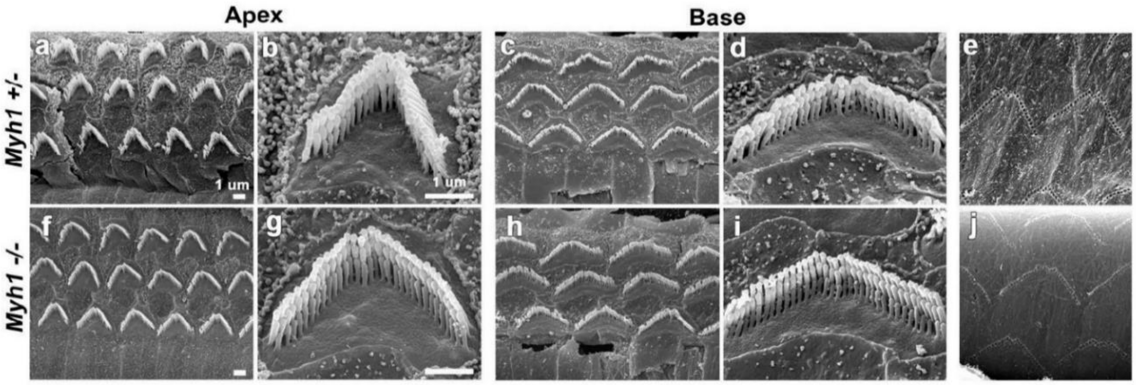

Supplementary Fig. 1. SEM images of the apical and basal cochlea of *Myh1*$^{+/+}$ and *Myh1*$^{-/-}$ mice. No clear difference in stereociliary bundles or tectorial membrane imprints were observed between *Myh1*$^{+/+}$ and *Myh1*$^{-/-}$ mice at postnatal day 30. Scale bars in 1 μm.

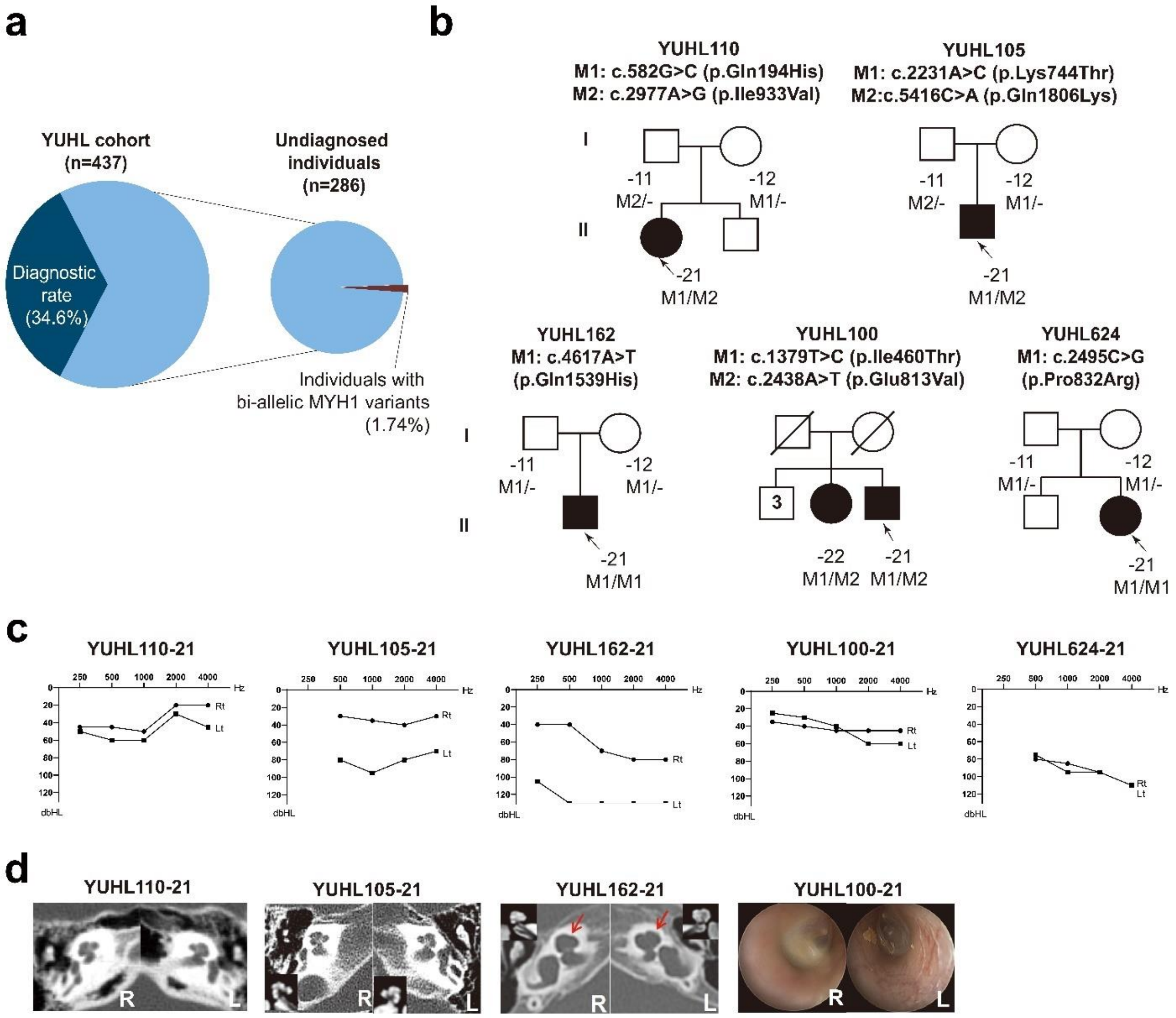

Supplementary Fig. 2. *MYH1* variants associated with non-progressive hearing loss. a Detection rate of individuals identified with bi-allelic variants of *MYH1* in the YUHL cohort. **b** Pedigrees of the five families in the cohort. **c** Audiometric characterization of the probands with *MYH1* variants. **d** Radiologic phenotypes of the probands with *MYH1* variants.

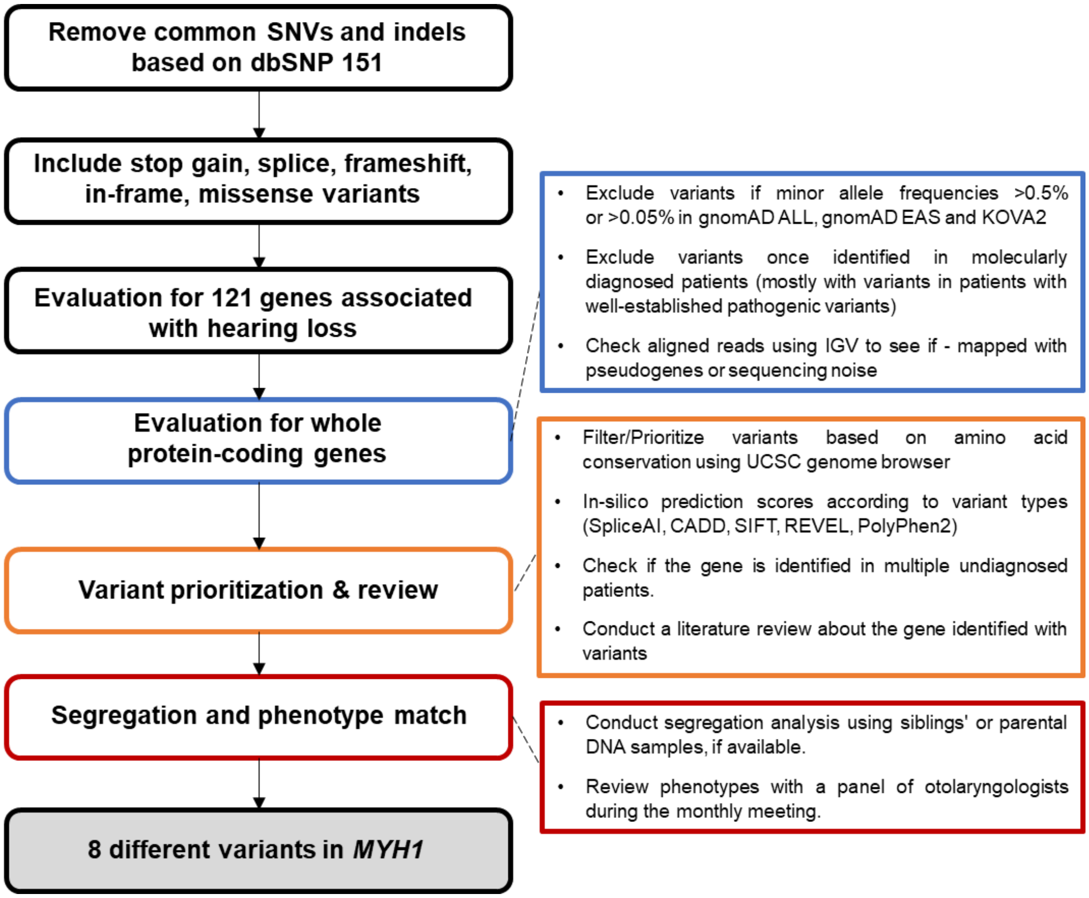

Supplementary Fig. 3. Exome sequencing data analysis pipeline for novel gene discovery.

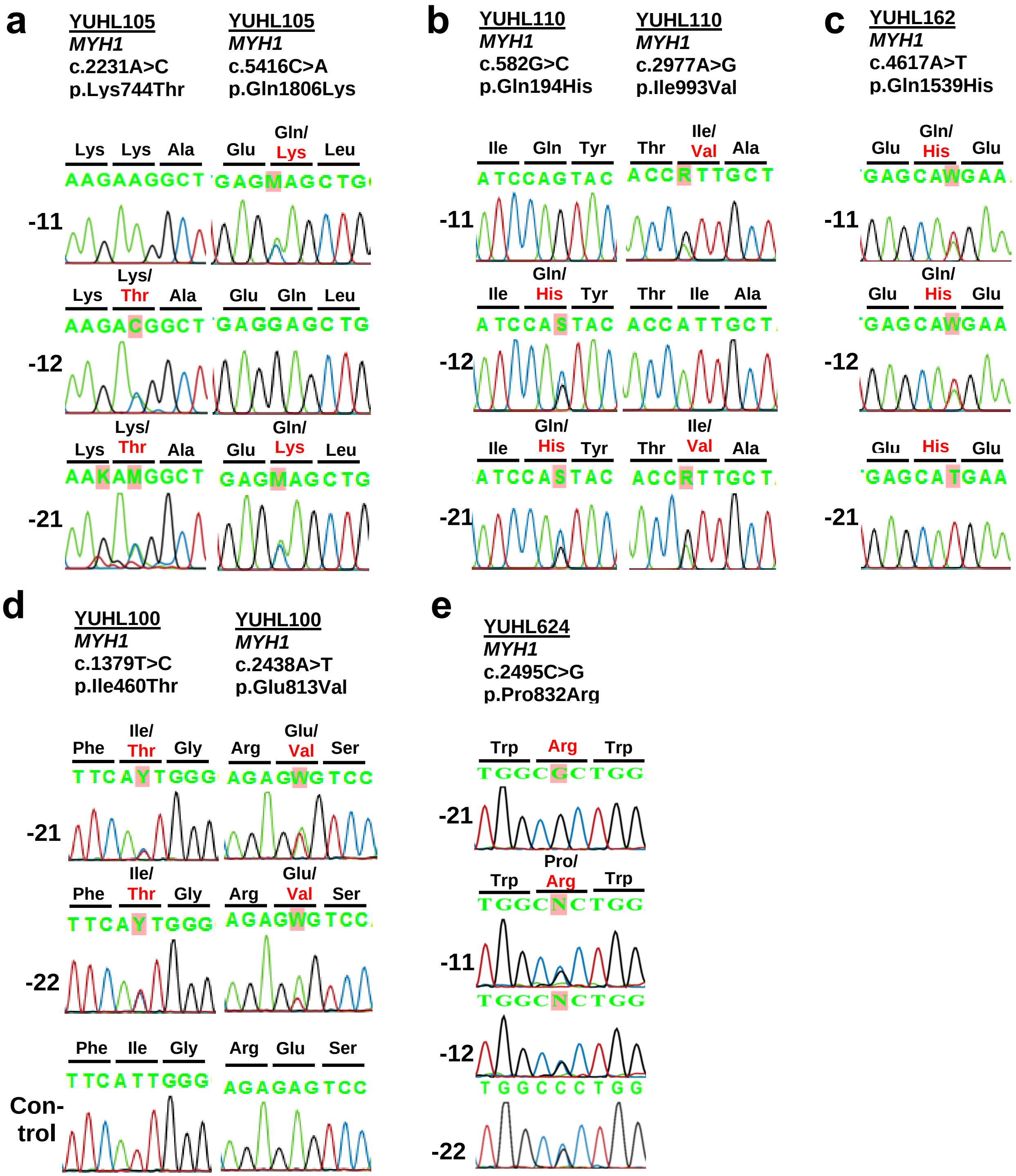

Supplementary Fig. 4. Sequence chromatogram of individuals with variants in *MYH1.*

(a–e) Eight variants of MYH1 were detected in five families with hearing loss. cDNA and amino acid change are indicated above. If no parental DNA is available, we used individual without hearing loss as control for PCR and sanger sequencing.

YUHL100-22

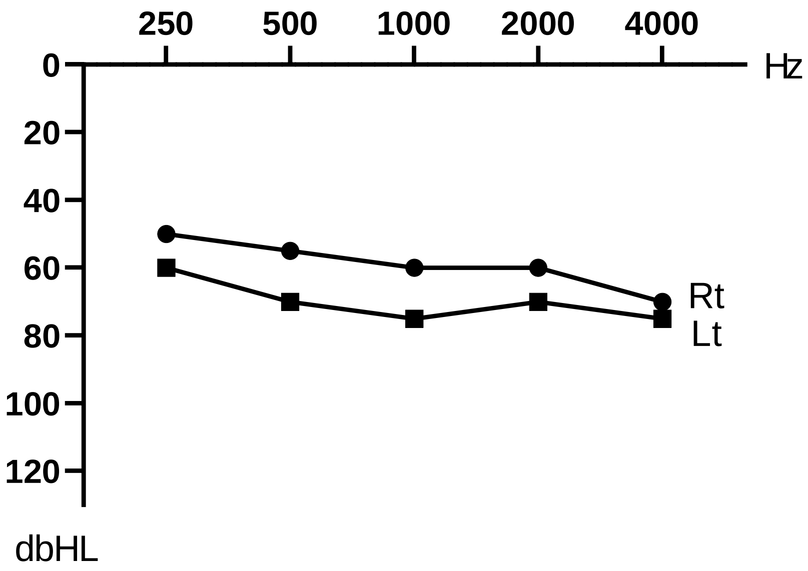

Supplementary Fig. 5. Pure-tone audiogram of YUHL100-22.

YUHL100-22 (75-year-old female) had non-progressive bilateral hearing loss with moderate-to-severe severity.

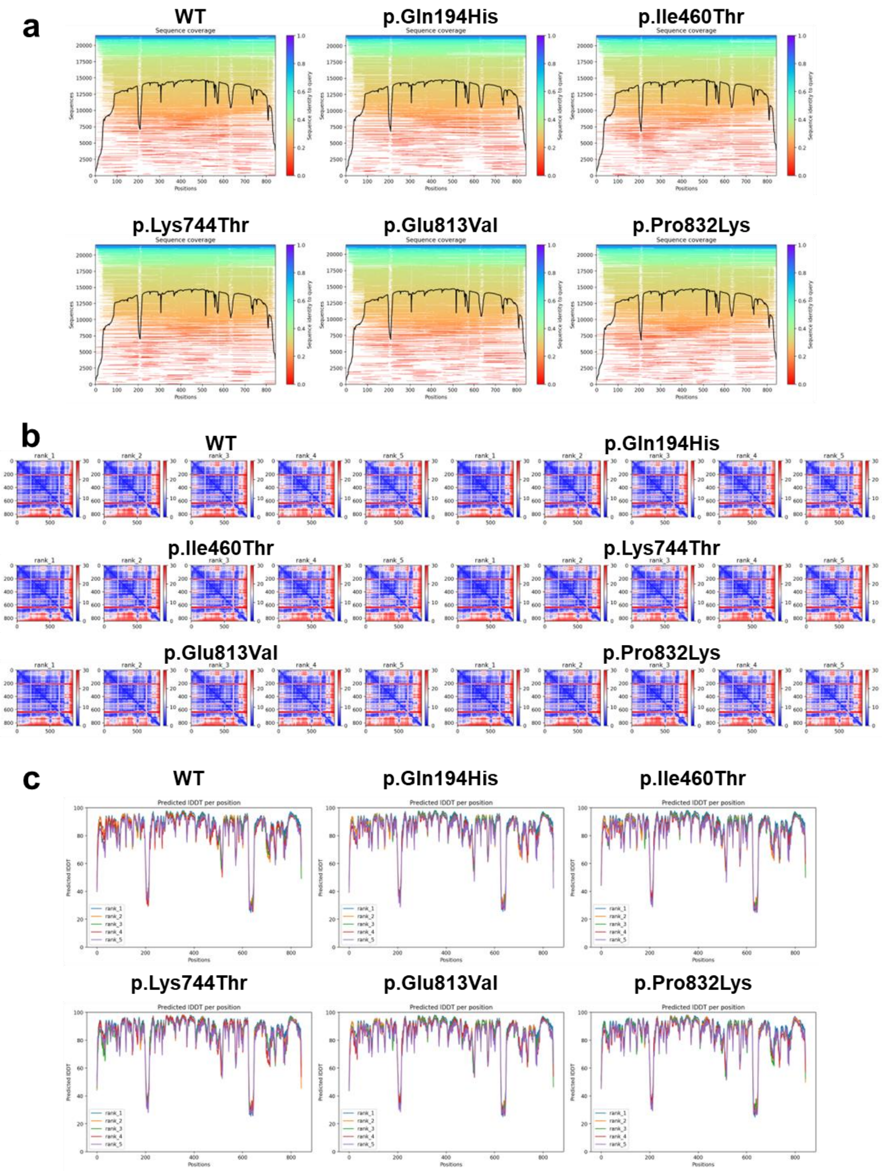

Supplementary Fig. 6. Prediction score of MYH1 proteins.

a Total sequence coverage, **b** Predicted Aligned Error (PAE) score, and **c** Local Distance Difference Test (LDDT) score calculated of WT and mutant MYH1 by ColabFold and AlphaFold2 prediction.

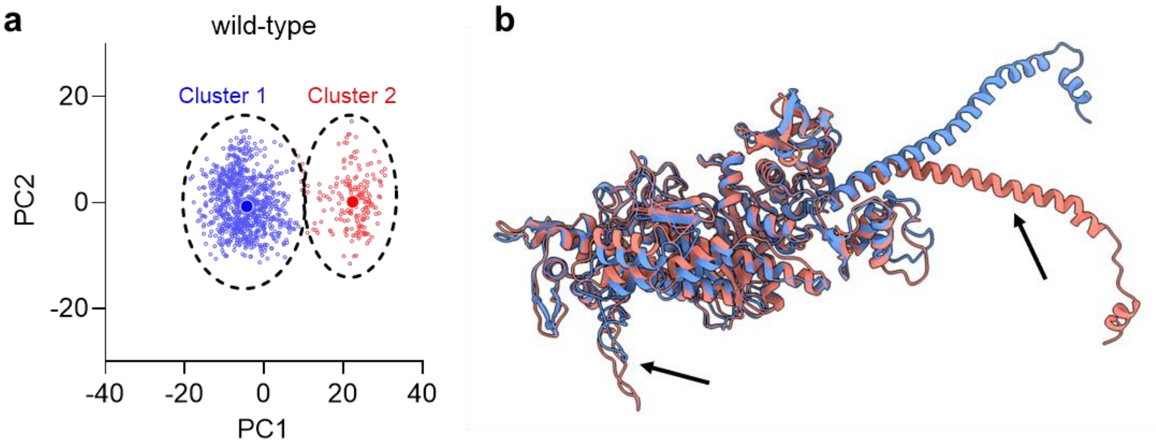

Supplementary Fig. 7. Cluster analysis of wild-type MYH1 MD simulation.

a Principal component analysis of wild-type MYH1 MD simulation. K-means clustering analysis showed two major clusters. Cluster centers are shown as enlarged points. **b** Comparison of structures from each cluster. Two major structural variations are pointed in arrows.

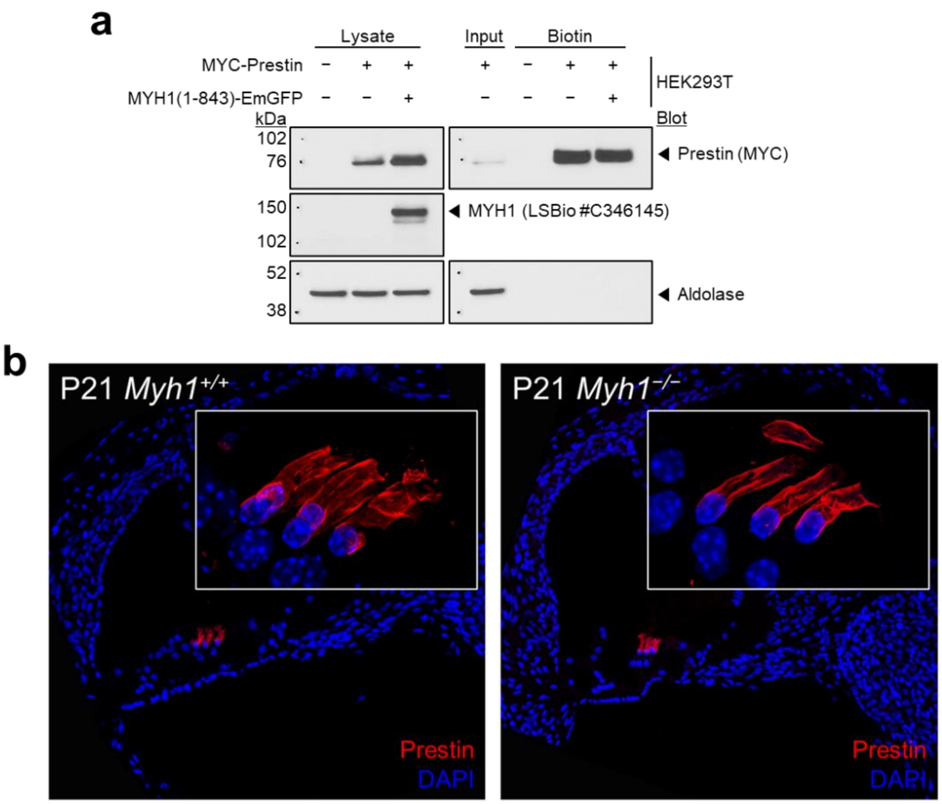

Supplementary Fig. 8. MYH1 does not affect Prestin localization.

a Surface biotinylation assay of Prestin in the absence or presence of MYH1. **b** Immunostaining of Prestin in *Myh1*$^{+/+}$ and *Myh1*$^{-/-}$ mice OHCs.

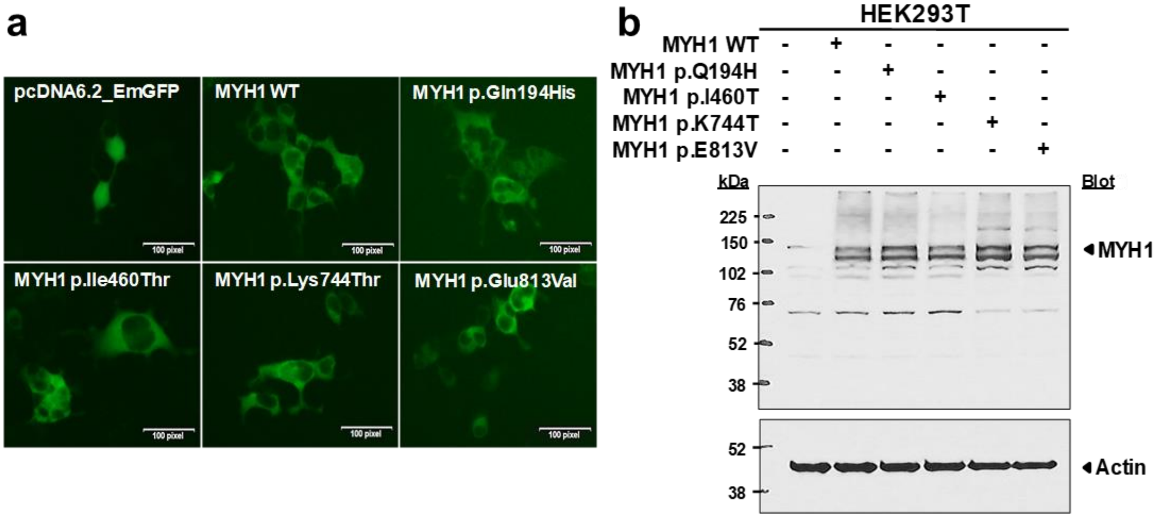

Supplementary Fig. 9. Expression of wild-type and variant MYH1 proteins in HEK293T cells.

a Expressed MYH1 protein corresponds to the N-terminal 843 of human MYH1 which include SRC Homology 3 (SH3) domain, myosin motor (MYSc) domain, and IQ calmodulin-binding motif. Distribution of MYH1 proteins was largely in cell body. **b** Immunoblotting of MYH1 variants. There was no significant difference in expression among transiently expressed wild-type and variant MYH1 proteins.

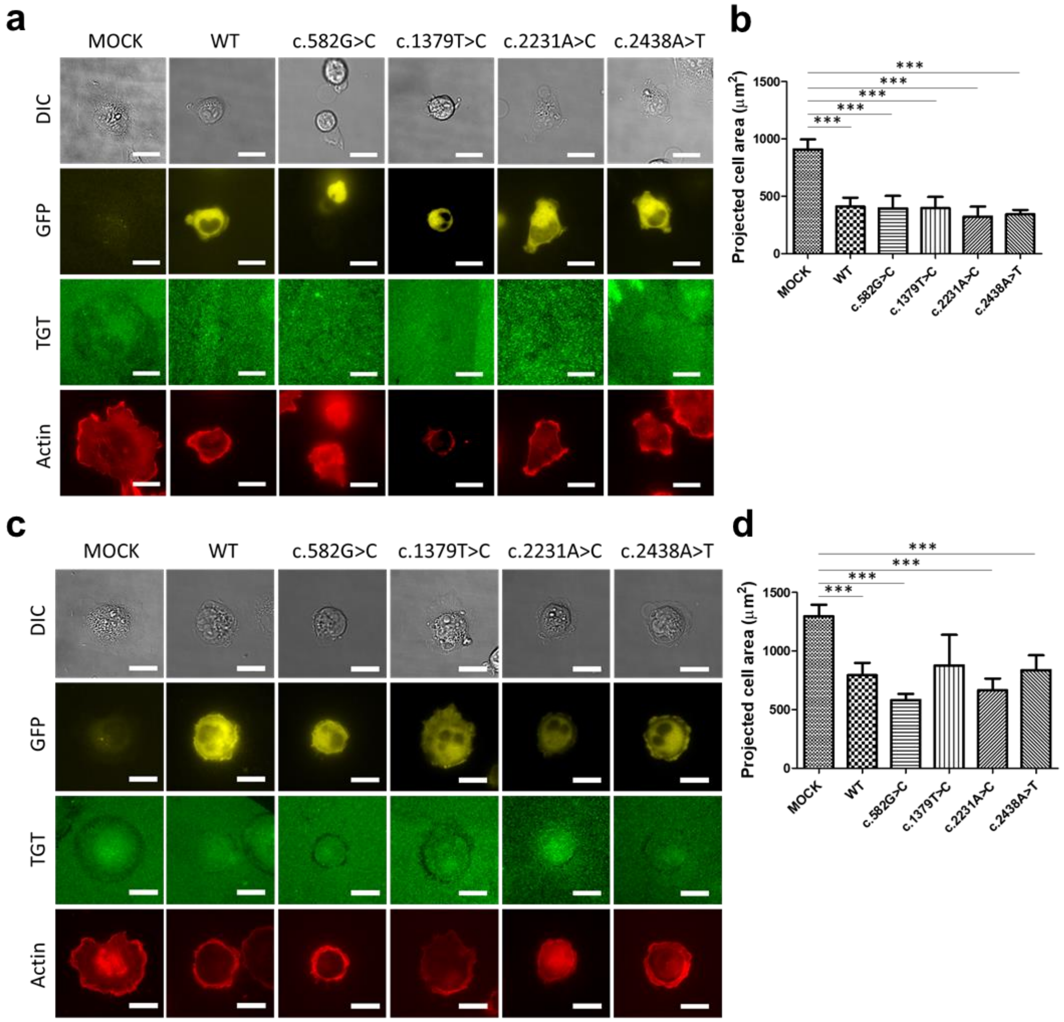

Supplementary Fig. 10. Functional consequences of *MYH1* variants in COS-7 cell.

a Initial cell adhesion on TGT surface (incubation time, t = 30 min). Adhesion of MYH1 wild-type or variant over-expressing COS-7 cells on 54pN TGT surface and TGT rupture patterns. Scale bar is 20 μm. **b** Measured projected area of MYH1 wild type or mutant over-expressing COS-7 cells on 54pN TGT surface (n = 12 to 27 individual cells). **c** Cell adhesion and spreading on TGT surface (incubation time, t = 1 hr). Adhesion of MYH1 wild-type or mutant over-expressing COS-7 cells on 54pN TGT surface and TGT rupture patterns. Fluorescence signal loss in the Cy3 channel was induced when a stronger molecular tension above 54 pN was applied on an integrin-cRGDfK bond. Scale bar is 20 μm. **d** Measured projected area of MYH1 wild type or mutant over-expressing COS-7 cells on 54pN TGT surface (n = 22 to 34 individual cells). The TGT assay in each condition was repeated at least three times. Data are presented as Mean ± SEM. *P<0.05; ** P < 0.01; *** P < 0.001; **** P < 0.0001.

Supplementary Table 1. Variants of *MYH1* found in individuals with hearing loss by whole exome sequencing.

ID	Sex	Age of onset	Nucleotide change	Amino acid change	Exon	Zygosity, segregation	dbSNP[a]	gnomAD[b]	KOVA2[c]	REVEL[d]	Classification[e]
**105-21**	M	Congenital	c.2231A>C	p.Lys744Thr	20	het, M	rs183833836	0.000144	0.00018882 (2/10592)	0.835	VUS (PM2_Supporting, PP3)
			c.5416C>A	p.Gln1806Lys	37	het, F	rs140873918	0.000306	0.00217926 (23/10554)	0.499	VUS (PM2_Supporting)
**110-21**	F	1st decade	c.582G>C	p.Gln194His	7	het, M	rs776053827	0.000024	0.00028339 (3/10586)	0.762	VUS (PM2_Supporting, PP3)
			c.2977A>G	p.Ile993Val	24	het, F	rs150191104	0.000277	0.003899562 (41/10514)	0.261	VUS (BS1_Supporting)
**162-21**	M	Congenital	c.4617A>T	p.Gln1539His	33	homo, M&F	rs3764850	0.000037	0.0009455371 (10/10576)	0.358	VUS (PM2_Supporting)
**100-21 -22**	M F	ND ND	c.1379T>C	p.Ile460Thr	14	ND	rs201061123	0.000277	-	0.882	VUS (PM2_Supporting, PP3)
			c.2438A>T	p.Glu813Val	22	ND	rs191339081	0.000314	0.00103950 (11/10582)	0.533	VUS (PM2_Supporting)
**624-21**	F	Congenital	c.2495C>G	p.Pro832Arg	22	homo, M&F	rs534110923	0.000509	0.003411029 (36/10554)	0.799	VUS (BS1_Supporting, PP3)

Abbreviations are as follows: ND, no data; M, heterozygous variant that is maternally inherited; F, heterozygous variant that is paternally inherited; M&F, homozygous variant.
[a] dbSNP database (http://www.ncbi.nlm.nih.gov/SNP).
[b] gnomAD browser (http://exac.broadinstitute.org/).
[c] AF archived from KOVA2 (the Korean Variant Archive 2; approximately n = 5,305).
[d] REVEL (rare exome variant ensemble learner) score predicting the deleteriousness of variants colored in red.
[e] Classifications of variants are based on VIP-HL (http://hearing.genetics.bgi.com/), an online platform for classifying variants according to the ACMG guidelines adapted for hearing loss. PM2_Supporting and BS1_Supporting are criteria given when the MAF of variants are in the range of 0.00007≤AF<0.0007 or 0.0007≤AF<0.003, respectively. For the reliable curation of variants identified in Korean, we utilized AF from the KOVA2 database as filtering AF for PM2 and BS1. PP3 is assigned for variants with a REVEL score above 0.7.

Supplementary Table 2. Other candidate genes in patients.

Individual	Gene	variant position (hg19)	Transcript	Nucleotide change	Amino acid change	Exon	dbSNP	gnomAD	KOVA2	REVEL	Result
**100-21**	*FAT3*	chr11:92531314_G/A	NM_001008781.2	c.5135G>A	p.Gly1712Glu	9	rs753513948	0.0002788	0.002308	0.288	identified as trans in affected sibling (100-22)
		chr11:92532352_G/A		c.6173G>A	p.Arg2058His	9	rs199620788	0.0003941	0.002405	0.26	
	ZRANB3	chr2:135960455_C/T	NM_032143.3	c.3088G>A	p.Gly1030Arg	20	rs746258198	0.00002893	0.00123	0.871	not segregated in 100-22
		chr2:135985381_C/A		c.2158+1G>T	-	14	rs76757575	0.00009516	0.000797	-	
	GTSE1	chr22:46725399_G/A	NM_016426.6	c.2071G>A	p.Asp691Asn	11	rs779875611	0.00003246	9.48E-05	0.368	not segregated in 100-22
		chr22:46725971_C/T		c.2164C>T	p.Gln722*	12	rs776761037	0.000004061	0.000571	-	
**105-21**	*FAM104B*	chrX:55172708_T/G	NM_138362.3	c.157A>C	p.Ile53Leu	3	rs1047034	0.00001142	-	0.098	hemizygous variant
**110-21**	*DNAH7*	chr2:196673555_G/C	NM_018897.2	c.9934C>G	p.Leu3312Val	53	rs200669357	0.0000583	0.000661	0.496	identified as cis, all inherited from unaffected mother
		chr2:196837160_T/C		c.1864A>G	p.Met622Val	16	rs780113708	0.0000082	-	0.129	
**162-21**	*SMS*	chrX:22002471_G/A	NM_004595.4	c.800G>A	p.Gly267Glu	8	-	-	-	0.649	OMIM-listed gene > Phenotype unmatched
	DMD	chrX:31515027_G/A	NM_004006.2	c.8425C>T	p.Arg2809Cys	57	rs143389016	0.00000575	-	0.324	OMIM-listed gene > Phenotype unmatched
**624-21**	*GSE1*	chr16:85688428_C/A	NM_014615.5	c.628C>A	p.Pro210Thr	5	rs563709852	0.0001181	0.000844	0.119	identified as cis, all inherited from unaffected mother
		chr16:85695115_-/GCCCGG		c.2014_2019dup	p.Gly672_Pro673dup	9	rs770052395	-	-	-	

Supplementary Table 3. Dual-energy X-ray absorptionmetry results.

Individual	Region	Area (cm^2)	Bone mineral content (g)	Bone mineral density (g/cm^2)	T-score	Z-score
105-21	Neck	3.52	1.62	0.461	-3.7	-1.6
	Troch	3.77	1.37	0.362	-3.3	
	Inter	7.71	4.23	0.548	-4.5	-1.1
	Total	15.01	7.22	0.481	-4.5	-1.7
110-21	Neck	5.26	3.93	0.747	-1.4	-1.4
	Troch	11.59	7.62	0.657	-0.5	
	Inter	22.9	20.95	0.915	-1.8	-1.7
	Total	39.76	32.5	0.817	-1.6	-1.6

Supplementary Table 4. Parameters used for simulating OHC circuit.

Variable	Value
EP	30 mV
G_{MET}	30 nS
x_0	20 nm
s_0	16 nm
s_1	35 nm
τ_{MET}	0.05 ms
$G_{K,f}$	1 ns
$G_{K,n}$	50 ns
$V_{h,K,f}$	−24 mV
$V_{h,K,n}$	−92 mV
$s_{K,f}$	6.4 mV
$s_{K,n}$	17 mV
E_K	−92 mV
$C_{lin,WT/KO}$	4 pF
α_{WT}	25.76 mV
α_{KO}	29.10 mV
$Q_{max,WT}$	3662 fC
$Q_{max,KO}$	1195 fC
$V_{h,Pres,WT}$	−37.02 mV
$V_{h,Pres,KO}$	−47.51 mV
